# Preparation of Mixed Bis-N-Heterocyclic Carbene Rhodium(I) Complexes

**DOI:** 10.3390/molecules27207002

**Published:** 2022-10-18

**Authors:** Ramón Azpíroz, Mert Olgun Karataş, Vincenzo Passarelli, Ismail Özdemir, Jesús J. Pérez-Torrente, Ricardo Castarlenas

**Affiliations:** 1 Departamento de Química Inorgánica—Instituto de Síntesis Química y Catálisis Homogénea (ISQCH), Universidad de Zaragoza—CSIC, C/Pedro Cerbuna 12, 50009 Zaragoza, Spain; 2 Department of Chemistry, Faculty of Sciences, Inonu University, 44280 Malatya, Turkey

**Keywords:** N-heterocyclic carbene, coumarin, rhodium, mixed-NHC, anagostic interactions

## Abstract

A series of mixed bis-NHC rhodium(I) complexes of type RhCl(η^2^-olefin)(NHC)(NHC’) have been synthesized by a stepwise reaction of [Rh(*μ*-Cl)(η^2^-olefin)_2_]_2_ with two different NHCs (NHC = N-heterocyclic carbene), in which the steric hindrance of both NHC ligands and the η^2^-olefin is critical. Similarly, new mixed coumarin-functionalized bis-NHC rhodium complexes have been prepared by a reaction of mono NHC complexes of type RhCl(NHC-coumarin)(η^2^,η^2^-cod) with the corresponding azolium salt in the presence of an external base. Both synthetic procedures proceed selectively and allow the preparation of mixed bis-NHC rhodium complexes in good yields.

## 1. Introduction

**Scheme 1:**
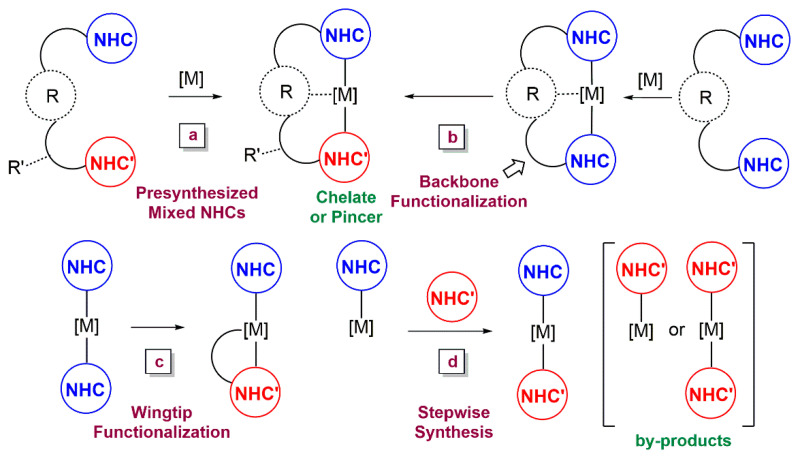
Conventional synthetic approaches to mixed bis-NHC metal complexes.

Since the pioneering work of Öfele [1] and Wanzlick [2] on the preparation of N-heterocyclic carbene (NHC) complexes and, particularly, that of Arduengo on the isolation of the first free NHC [3], the interest around these species in organometallic catalysis and beyond has grown exponentially [4,5,6,7,8]. The relatively easy synthesis enables fine tuning of their electronic and steric properties [9], which nowadays places them as the ligand of choice in many transition metal-mediated applications. Moreover, the introduction of a second carbene molecule in bis-NHC complexes have resulted in a synergic enhancement of the benefits imparted by these ligands in inter alia catalytic applications [10], the activation of small molecules [11], biomedicine [12] or luminescence [13]. Although less common, the presence of two different NHC moieties provides complementary reactivity to complexes bearing identical NHC ligands. The synthesis of these mixed bis-NHC species is not trivial due to competitive substitution reactions or the formation of isoleptic bis-NHC byproducts. To circumvent these problems, pre-synthesized unsymmetrical precursors have been used for the preparation of chelated or pincer heteroleptic bis-NHC derivatives [14,15,16,17,18,19] (Scheme 1a). Another approach involves additional functionalization after the pre-synthesis of symmetrical complexes, either within the bis-NHC backbone for multitopic ligands [20,21,22] (Scheme 1b), or on the wingtip of a monodentated NHC [23,24,25] (Scheme 1c). On the other hand, mixed monodentate bis-NHC derivatives with a variety of metals have been prepared by using a stepwise methodology [26,27,28,29,30,31,32,33,34,35,36,37,38,39,40,41,42,43,44,45] (Scheme 1d). The dissimilar stereoelectronic properties of both carbenes, such as in expanded-ring NHCs [37], mesoionic (MIC) [38,39,40] or acyclic carbenes [41,42,43], make the control of selectivity more accessible. Moreover, alternative synthetic protocols such as microwave irradiation [44] or mechanochemistry [45] have also proven to be effective.

Heteroleptic bis-NHC complexes have in some cases overcome the performances of isoleptic bis-NHC counterparts in many applications. For example, various mixed bis-NHC iridium [34], platinum [35], gold [36], or silver [45] complexes present promising activity as anticancer therapeutics. In material science, polystyrene-linked bis-NHC copper(I) complexes have shown application as sensors [46], whereas cyclometalated iridium (III) derivatives containing mixed NHC-acyclic carbene are top-performing emitters in organic light-emitting diodes (OLEDs) [42]. Regarding organometallic catalysis, the most relevant examples are developed for ruthenium-promoted olefin metathesis [28,41], but also for epoxide isomerization [17], hydrogen borrowing transformations [18], cross-coupling reactions [30], olefin hydrogenation [31], or azide–alkyne cycloadditions [32]. It is interesting to note the application of mixed bis-NHC palladium complexes for a reliable determination of the electronic properties of newly synthesized NHC via ^13^C NMR spectroscopy, coined as the Huynh’s Electronic Parameter (HEP) [47].

Mixed monodentate bis-NHC rhodium(I) complexes are quite scarce [26,27]. They have been prepared from dinuclear Rh-NHC precursors by CO- or chlorido-bridge cleavage with different NHC’ moieties, being the marked difference in the stereolectronic properties of both carbenes the key for success. In this context, our research group has focused on the development of rhodium-NHC complexes and their applications in catalytic processes [48,49,50,51,52]. In particular, the dinuclear compounds [Rh(μ-Cl)(NHC)(η^2^-olefin)]_2_ are useful starting materials for the preparation of a great variety of mononuclear complexes of type RhCl(NHC)(η^2^-olefin)(L) by simple bridge-cleavage with a nucleophilic ligand, which have proven to be efficient catalysts for *gem*-selective alkyne dimerization [48,51], hydrothiolation [50], or hydropyridonation [52]. We envisaged that nitrogen-centered nucleophiles could be substituted by carbon-centered NHCs, giving access to mixed bis-NHC species. Alternatively, we have synthesized a series of imidazolium salts functionalized with a pendant coumarin group, that have allowed for the synthesis of coumarin-functionalized NHC rhodium complexes by reaction with the internal base in the dinuclear precursor [Rh(*μ*-OCH_3_)(η^2^,η^2^-cod)]_2_ (cod = 1,5-cyclooctadiene) [53]. In the course of our investigation, we observed that in the presence of an external base, pentacoordinated bis-coumarin-NHC rhodium derivatives were obtained. Thus, we reasoned that a stepwise reaction using different azolium salt precursors could pave the way to mixed bis-coumarin-functionalized NHC complexes.

## 2. Results and Discussion

**Scheme 2:**
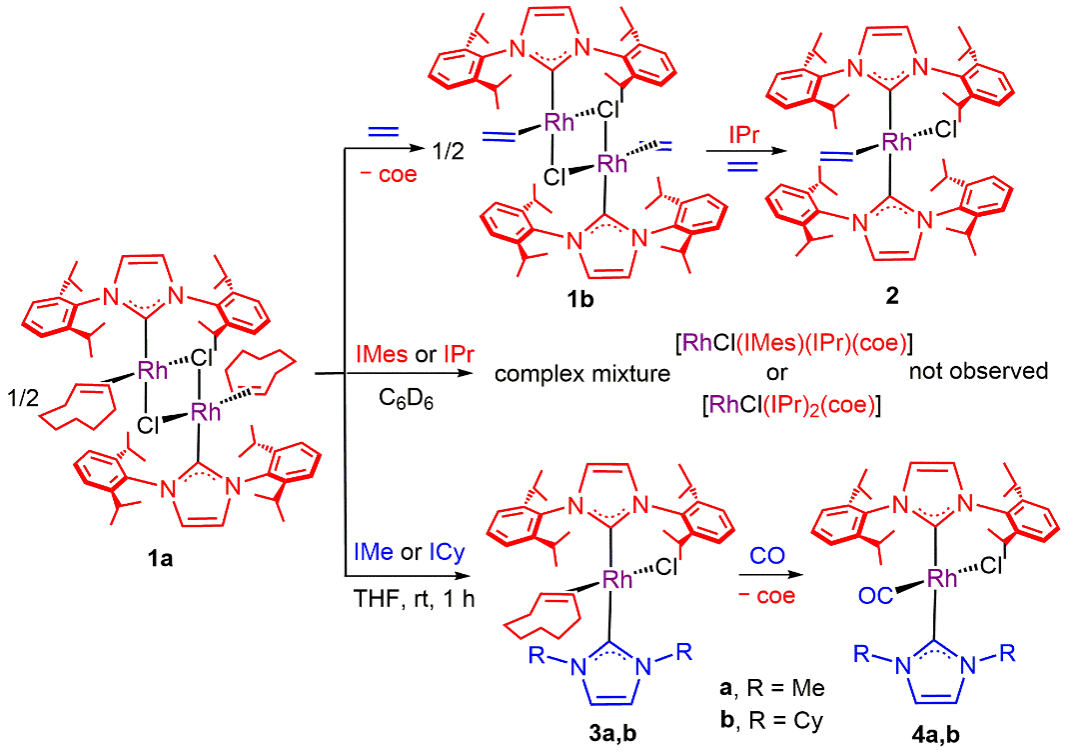
Steric relief as a key factor for the preparation of bis-NHC rhodium(I) complexes.

The James’s dimer [Rh(μ-Cl)(η^2^-coe)(IPr)]_2_ {coe = cyclooctene, IPr = 1,3-bis(2,6-diisopropylphenyl)imidazolin-2-carbene} (**1a**) [54] is a suitable precursor for the stepwise synthesis of mixed bis-NHC rhodium(I) complexes. However, initial attempts to introduce a carbene displaying stereoelectronic properties similar to IPr, such as IMes {IMes = 1,3-bis(1,3,5-trimethylphenyl)imidazolin-2-carbene}, were unsuccessful. The addition of free IMes to a C_6_D_6_ solution of **1a** at room temperature resulted in a complex mixture of products, including Rh-hydride species [23], in which traces of the substitution derivative [Rh(*μ*-Cl)(η^2^-coe)(IMes)]_2_ could be detected (Scheme 2). It is likely that the high steric hindrance imparted by both bulky NHCs, in addition to the pseudo-spherical coe ligand, could hamper the formation of the putative mixed-NHC species RhCl(η^2^-coe)(IMes)(IPr). However, the steric relief imparted by the less encumbering ethylene ligand compared to coe [55], allowed the Tilset’s group to prepare the bis-IMes compound RhCl(η^2^-ethylene)(IMes)_2_ [56]. Accordingly, the related IPr derivative RhCl(η^2^-ethylene)(IPr)_2_ (**2**) can now be prepared. As described previously by James et al. [54], the dinuclear Rh-IPr-coe precursor **1a** does not react with an excess of free IPr, but **2** is cleanly formed by the reaction of [Rh(μ-Cl)(η^2^-ethylene)(IPr)]_2_ (**1b**) [57] with one equivalent of free IPr under an ethylene atmosphere.

The NMR data of **2** agree with the presence of two carbene ligands in a mutual trans disposition. In the ^1^H NMR spectrum, the characteristic signals of IPr integrate in a double ratio with respect to that of ethylene, which appears as a singlet at δ 2.01 ppm in agreement with the C*_2v_* symmetry of the complex. The appearance of two septuplets at 3.51 and 2.76 ppm, corresponding to the CH-isopropyl protons of the IPr, indicates a restricted rotation of the carbene ligands around the Rh-C axis [49]. In the ^13^C{^1^H}-APT spectrum, the most significant feature is the downfield shift of the carbene carbon atom to δ 191.8 ppm related to **1b** (179.7 ppm), reflecting the increase of electron density at the metal center as a result of the coordination of a second powerful electron releasing IPr ligand [58]. Moreover, the reduction of the rhodium-carbon coupling (*J*_Rh-C_ = 41.6 Hz vs. 62.3 Hz in **1b**) is a consequence of an opposite disposition of two ligands with a strong trans influence.

**Figure 1:**
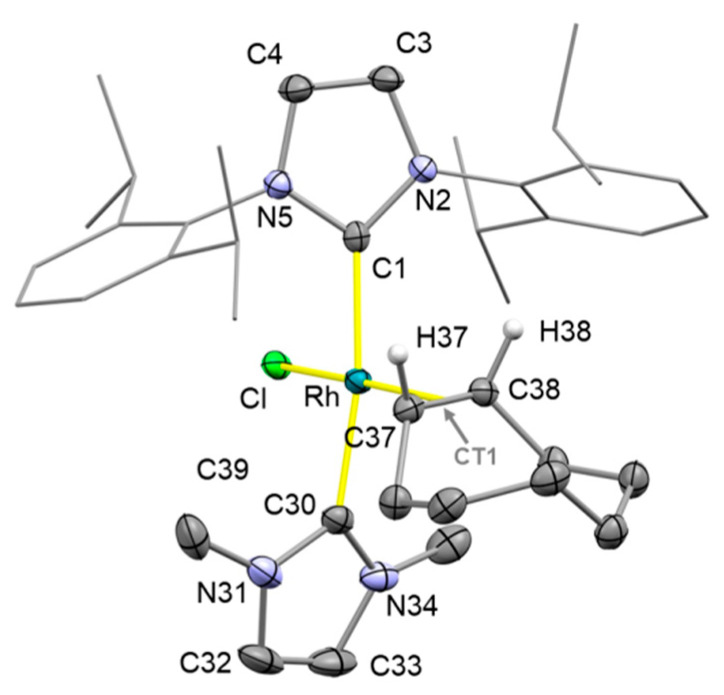
ORTEP view of **3a**. For clarity, most hydrogen atoms are omitted and selected groups are shown in a wireframe style. Thermal ellipsoids are at 50% probability. Selected bond lengths (Å) and angles (°) are: Rh-C1 2.061(2), Rh-C30 2.030(2), Rh-Cl 2.4215(6), Rh-CT1 2.0050(2), C37-C38 1.405(3), C1-Rh-C30 169.65(9), C30-Rh-Cl 82.13(7), C1-Rh-Cl 89.07(6), CT1-Rh-Cl 169.320(18). CT1, centroid of C37 and C38. IPr: pitch angle (θ) 5.1, yaw angle (ψ) 4.1; Ime: pitch angle (θ) 9.3, yaw angle (ψ) 2.1.

The steric pressure over the η^2^-olefin within a rhodium-bis-IPr architecture has been harnessed previously by Crudden et al., who described its facile decoordination under a nitrogen atmosphere to yield dinitrogen adducts [59]. Unfortunately, the mixed bis-NHC derivative RhCl(η^2^-ethylene)(IMes)(IPr) could not be cleanly obtained by the addition of IMes to **1b**. In addition, attempts to use diolefin precursors of the type RhCl(η^2^,η^2^-cod)(NHC) failed [60]. More recently, the group of Chaplin has disclosed that bis-NHC rhodium complexes bearing a η^2^-coe ligand could be prepared with a less sterically demanding IBiox carbene [61]. According to this, the treatment of **1a** with less-bulky NHCs, such as IMe or ICy, prepared in situ by deprotonation of the corresponding azolium salts, resulted in the successful formation of the mixed bis-NHC complexes RhCl(η^2^-coe)(IPr)(NHC) (**3**) {NHC = 1,3-dimethylimidazolin-2-carbene (IMe) (**3a**) and 1,3-dicyclohexylimidazolin-2-carbene (ICy) (**3b**)}, which were isolated as yellow solids in good yields (Scheme 2). The η^2^-olefin ligand of complexes **3** remains coordinated in solution at room temperature, but can be smoothly replaced by CO to yield the carbonyl complexes RhCl(CO)(IPr)(NHC) (NHC = IMe, **4a**; ICy, **4b**). Single crystals of **3a** suitable for X-ray structure determination were grown by slow diffusion of hexane into a saturated toluene solution of the complex. Figure 1 shows the molecular structure of **3a** and the selected bond lengths and angles.

The crystal structure of **3a** shows a distorted square planar geometry at the metal center, with a trans disposition of the NHC ligands [C1-Rh-C30 169.65(9)°]. The Rh-C_NHC_ bond lengths {2.061(2) Å for IPr and 2.030(2) Å for IMe} are slightly longer than that found in similar mono-NHC Rh^I^-Cl-coe complexes {1.956(2) Å in [Rh(μ-Cl)(η^2^-coe)(IPr)]_2_ [60], 1.986(4) Å in RhCl(η^2^-coe)(IPr)(pyridine) [57], and 1.9835(18) Å in RhCl(η^2^-coe)(IPr)(2-pyridylacetonitrile) [49]}, reflecting the high trans influence of NHCs. The coordinated olefinic bond C37-C38, as well as the NHC cores of the IPr and IMe ligands, lie almost perpendicular to the coordination plane Rh-Cl-C1-C30-CT1 (79.6°, 85.3°, 69.6°, respectively). In addition, the pitch and yaw angles observed for each NHC ligand indicate a moderately distorted coordination to the metal center with respect to the corresponding rhodium-carbon bond.

**Figure 2:**
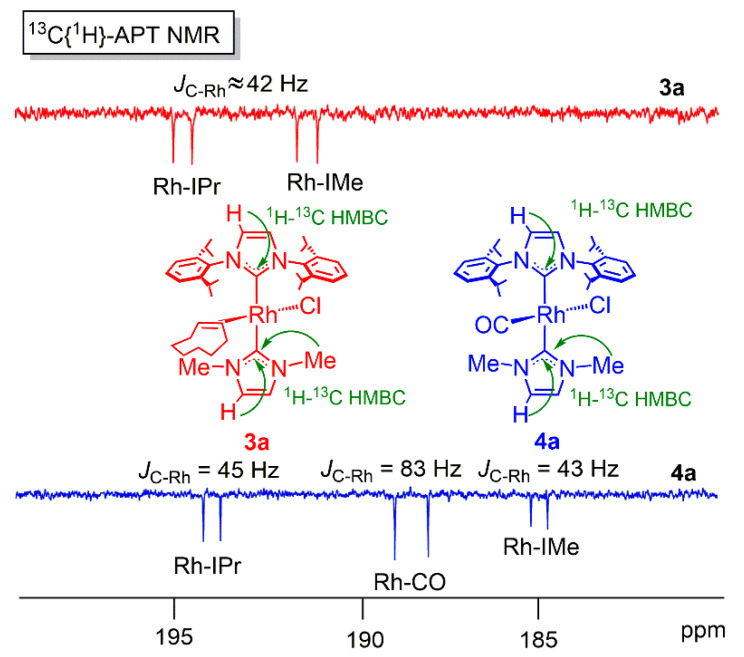
Downfield area of the ^13^C{^1^H}-APT NMR spectra of **3a** and **4a**.

The low temperature NMR data for complexes **3** agree with the structure reported in the solid state for **3a**. Thus, in the ^1^H NMR spectrum at 243 K, two septuplets ascribed to the CH-isopropyl protons of the IPr are observed around δ 4.3 and 2.7 ppm, which broaden at room temperature due to a Rh-IPr rotational process (see Figure S1 in Supporting Information). The presence of different NHC ligands in **3** is confirmed by the appearance of two resonances for the =CHN heterocyclic protons around δ 6.5 (IPr), 5.93 (IMe, **3a**), and 6.34 ppm (ICy, **3b**). In addition, two relatively deshielded doublets are displayed in the ^13^C{^1^H}-APT spectrum around δ 194 (IPr), 191.4 (IMe, **3a**), and 188.0 (ICy, **3b**), with relatively short couplings of around 42 Hz, as commented for **2**. The presence of η^2^-olefin ligands is corroborated by a multiplet around δ 3 ppm in the ^1^H NMR, and a doublet around 54 ppm (*J*_C-Rh_ = 16 Hz) in the ^13^C{^1^H}-APT spectra. The substitution of coe by carbon monoxide in **4** results in the appearance of a new doublet in the ^13^C{^1^H}-APT spectrum. Resonance assignment is facilitated by ^1^H-^13^C HMBC correlation peaks between the imidazolinyl protons and the corresponding carbene-carbon atoms within NHC moieties (Figure 2). As expected, the activation barrier for IPr-Rh rotation is reduced for carbonyl complexes **4** [49]. Therefore, only one septuplet for CH-isopropyl protons is observed around δ 3.4 ppm.

**Scheme 3:**
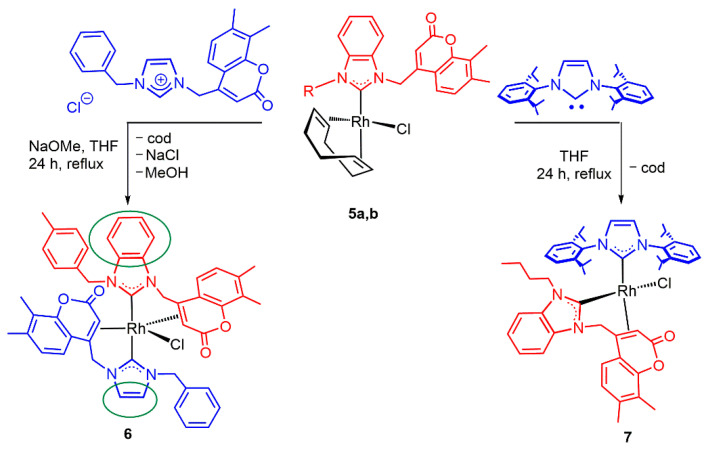
Preparation of coumarin-functionalized mixed bis-NHC rhodium complexes.

**Figure 3:**
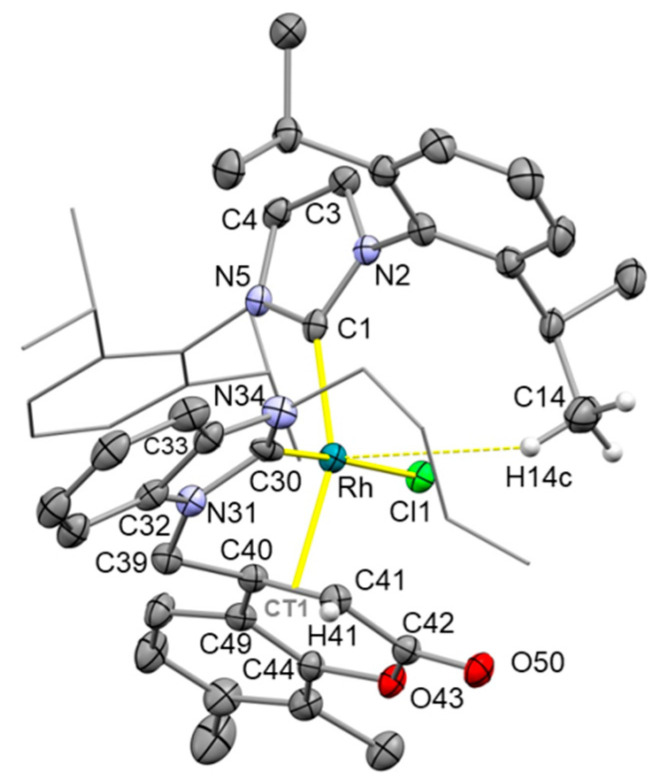
ORTEP view of **7**. For clarity, most hydrogen atoms are omitted and selected groups are shown in a wireframe style. Thermal ellipsoids are at 50% probability. Selected bond lengths (Å) and angles (°) are: Rh-Cl1 2.4116(8), Rh-C1 2.070(3), Rh-C30 1.959(3), Rh-CT1 2.0294(3), C40-C41 1.428(4), C30-Rh-C1 98.57(12), C1-Rh-Cl1 85.11(8), C30-Rh-Cl1 175.78(9), CT1-Rh-Cl1 97.55(2), CT1-Rh-C1 153.84(9), C30-Rh-CT01 80.07(9). IPr: pitch angle (θ) 12.7, yaw angle (ψ) 3.4; BzICou^Bu^: pitch angle (θ) 0.5, yaw angle (ψ) 11.1. CH···Rh contact: Rh·H14c 3.06(4), C14-H14c 0.90(4), Rh·C 3.774(4), Rh-H14c-C14 138(3).

Wingtip functionalized NHCs ligands can also participate in the formation of mixed bis-NHC rhodium(I) complexes. In this context, coumarin-functionalized-NHC metal complexes display interesting catalytic and luminescence properties [62,63,64,65,66]. In particular, we have prepared coumarin-NHC derivatives of type RhCl(NHC-Cou)(η^2^,η^2^-cod) (**5**) in which, in contrast to the behavior observed for related IPr- or IMes-cod compounds, the diolefin ligand can be replaced in the presence of an excess of carbene to yield bis-NHC complexes [53]. Thus, a stepwise reaction using different carbene precursors enabled the synthesis of mixed-NHC species (Scheme 3). In this way, an imidazole-benzimidazole mixed-NHC complex RhCl(κ*C*,η^2^-BzICou^tol^)(κ*C*,η^2^-ICou^Bz^) {BzICou^tol^ = 1-(4-methylbenzyl)-3-(7,8-dimethyl-2*H*-chromen-2-one-4-yl)benzimidazolin-2-carbene, ICou^Bz^ = 1-(benzyl)-3-(7,8-dimethyl-2*H*-chromen-2-one-4-yl)imidazolin-2-carbene} (**6**) was obtained by refluxing a THF solution of the precursor RhCl(BzICou^tol^)(η^2^,η^2^-cod)] (**5a**) and the azolium salt [HICou^Bz^]Cl in the presence of sodium methoxide for 24 h. Under similar reaction conditions, RhCl(BzICou^Bu^)(IPr) (**7**) was formed starting from RhCl(BzICou^bu^)(η^2^,η^2^-cod) (**5b**) and free IPr. Although the solid state structure of **6** could not be determined by X-ray diffraction methods, a trigonal bipyramidal structure with a trans disposition of the NHC ligands is assumed in an analogy to the related coumarin-functionalized bis-NHC complexes RhCl(BzICou^R^)_2_ and RhCl(ICou^R^)_2_ described previously [53]. Interestingly, the X-ray single-crystal analysis of the mixed bis-NHC compound **7** revealed a cis disposition for the IPr and coumarin-BzI carbenes, an uncommon feature in square planar rhodium(I) complexes when a trans configuration is feasible [67,68] (Figure 3).

The crystal structure of **7** exhibits a severely distorted square planar coordination geometry at the metal center with an Rh·H-C short contact. In fact, the NHC moieties adopt a cis disposition [C30-Rh-C1 98.57(12)°] with the chlorido ligand lying trans to C30 from the BzICou^Bu^ ligand [C30-Rh-Cl1 175.78(9)°]. On the other hand, the angle CT1-Rh-C1 [153.84(9)°] between C1 from the IPr ligand and the centroid of the coordinated olefinic bond C40-C41 of the coumarin moiety is significantly smaller than the ideal value for a trans disposition. In addition, the short Rh···H14c-C14 [3.06(4) Å] contact is observed [r_vdW_(H) + r_vdw_(Rh) ≈ 3.2 Å] [69], possibly indicating the presence of an anagostic interaction between one IPr methyl group and the metal center [70]. The Rh-C_NHC_ distances are quite different {1.959(3) Å for BzI-Cou vs. 2.070(3) Å for IPr}, likely as a consequence of the higher trans influence of the olefin related to chlorido ligand. As for the bidentate BzICou^Bu^ ligand, its reduced bite angle [80.07(9)°] brings about a severe deviation of the NHC core from the ideal arrangement with respect to the Rh-C30 bond (yaw angle, ψ 11.1°), similar to what is already observed in the related bis-chelate complexes RhCl(BzICou^R^)_2_ and RhCl(ICou^R^)_2_ [71].

**Figure 4:**
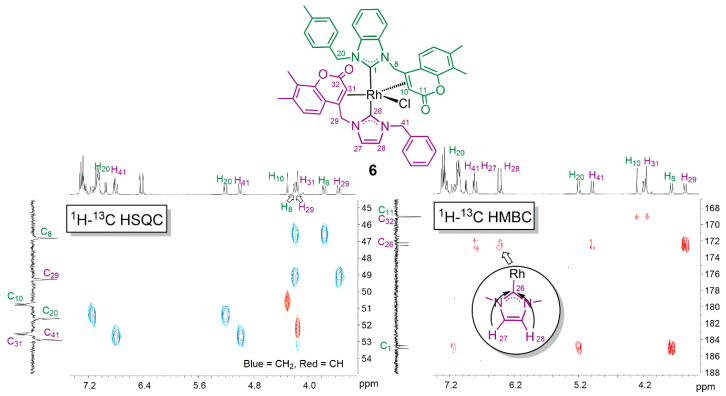
Selected areas of 2D-NMR correlation spectra for **6**: ^1^H-^13^C HSQC (**left**) and ^1^H-^13^C HMBC (**right**).

The presence of two different NHC ligands in **6** and **7** is confirmed by the NMR data. The ^13^C{^1^H}-APT NMR spectrum of **6** display two doublets at δ 184.8 and 172.4 ppm with Rh-C coupling constants around 33 Hz, ascribed to carbene carbon atoms. Correlations of the imidazolinyl protons at δ 6.45 and 6.40 ppm with the carbon signal at 172.4 ppm in the ^1^H-^13^C-HMBC spectrum of **6** helps to ascribe that signal to the I-Cou ligand (Figure 4). In addition, four doublets with *J*_H-H_~13 Hz are observed for the N-methylene protons in the ^1^H NMR spectrum. The coordination of the olefinic group of coumarin moieties is reflected in the appearance of high field doublets at 72.0 ppm (*J*_C-Rh_ = 12.1 Hz) and 50.8 ppm (*J*_C-Rh_ = 7.2 Hz) for BzI-Cou and 74.0 ppm (*J*_C-Rh_ = 11.7 Hz) and 52.5 ppm (*J*_C-Rh_ = 7.6 Hz) ascribed to the I-Cou ligand. Regarding 7, the presence of two different NHC ligands is corroborated by the observation of two low field doublets at 196.1 ppm (*J*_C-Rh_ = 52.2 Hz) and 191.1 ppm (*J*_C-Rh_ = 60.2 Hz) for BzI-Cou and IPr, respectively, in the ^13^C{^1^H}-APT NMR spectrum. The deshielding of these resonances compared to those of **6** could be ascribed to a more electron-rich rhodium center due to the coordination of only one electron-acceptor olefin ligand, whereas the higher *J*_C-Rh_ values reflect Rh-C shorter separations.

## 3. Materials and Methods

All reactions were carried out with the rigorous exclusion of air and moisture, using Schlenk-tube techniques and a dry box when necessary. The reagents were purchased from commercial suppliers and used as received. Organic solvents were dried by standard procedures and distilled under argon prior to use, or obtained oxygen- and water-free from a Solvent Purification System (Innovative Technologies). Deuterated solvents were deoxygenated and dried over sodium metal (C_6_D_6_ and toluene-*d*_8_) or activated molecular sieves (CDCl_3_). The organometallic precursors [Rh(μ-Cl)(η^2^-coe)(IPr)]_2_ (**1a**) [54], [Rh(μ-Cl)(η^2^-ethylene)(IPr)]_2_ (**1b**) [57], and RhCl)(η^2^,η^2^-cod)(NHC-Cou) (**5**) [53] were prepared as previously described in the literature. Chemical shifts (expressed in parts per million) are referenced to residual solvent peaks. Coupling constants, *J*, are given in Hz. Spectral assignments were achieved by a combination of ^1^H-^1^H COSY, ^13^C{^1^H}-APT and ^1^H-^13^C HSQC/HMBC experiments. The attenuated total reflection infrared spectra (ATR-IR) of solid samples were run on a PerkinElmer Spectrum 100 FT-IR spectrometer. C, H, and N analyses were carried out in a Perkin–Elmer 2400 CHNS/O analyzer. High-resolution electrospray mass spectra (HRMS) were acquired using a MicroTOF-Q hybrid quadrupole time-of-flight spectrometer (Bruker Daltonics, Bremen, Germany).

**Preparation ofRhCl(η^2^-CH_2_=CH_2_)(IPr)_2_ (2).** A solution of complex **1b** (300 mg, 0.27 mmol) and free IPr (230 mg, 0.59 mmol) in 50 mL of toluene was treated with 2 bars of ethylene and stirred for 1 h at room temperature. After filtration through Celite, the solvent was evaporated to dryness. The addition of n-hexane induced the precipitation of a yellow solid, which was washed with n-hexane (3 × 4 mL) and dried in vacuo. Yield (392 mg, 77%). HRMS (ESI) *m*/*z* Calcd for RhC_54_H_72_N_4_ (M-C_2_H_4_-Cl): 879.4807. Found: 879.4840. ^1^H NMR (400.1 MHz, C_6_D_6_, 298 K): δ 7.28 (t, *J*_H-H_ = 7.9, 4H, H_p-IPr_), 7.20 and 7.06 (both d, *J*_H-H_ = 7.9, 8H, H_m-IPr_), 6.34 (s, 4H, =CHN), 3.51 and 2.76 (both sept, *J*_H-H_ = 7.1, 8H, CHMe_IPr_), 2.01 (d, *J*_H-Rh_ = 2.0, 4H, CH_2_=CH_2_), 1.27, 1.08, 0.98, and 0.96 (all d, *J*_H-H_ = 7.1, 48H, CHMe_IPr_). ^13^C{^1^H}-APT NMR (75.5 MHz, C_6_D_6_, 298 K): δ 191.8 (d, *J*_C-Rh_ = 41.6, Rh-C_IPr_), 148.4 and 145.2 (both s, C_q-IPr_), 138.2 (s, C_q_N), 129.1 (s, CH_p-IPr_), 124.3 and 123.0 (both s, CH_m-IPr_), 123.9 (s, =CHN), 38.8 (d, *J*_C-Rh_ = 15.7, CH_2_=CH_2_), 28.8 and 28.5 (both s, CHMe_IPr_), 26.3, 26.2, 23.2, and 22.9 (all s, CHMe_IPr_). Figure S2: 1H NMR spectrum of 2 in C6D6 at 298 K. Figure S3. 13C{1H}-APT NMR spectrum of 2 in C6D6 at 298 K. Figure S4. 1H-1H COSY NMR spectrum of 2 in C6D6 at 298 K. Figure S5. 1H-13C HSQC NMR spectrum of 2 in C6D6 at 298 K. Figure S6. 1H-13C HMBC NMR spectrum of 2 in C6D6 at 298 K.

**Preparation ofRhCl(η^2^-coe)(IMe)(IPr) (3a).** A solution of dinuclear complex **1a** (300 mg, 0.23 mmol) in 50 mL of toluene was treated with a solution of IMe (50 mg, 0.51 mmoles) in THF and stirred for 1h at room temperature. After filtration through Celite, the solvent was evaporated to dryness. The addition of n-hexane induced the precipitation of a yellow solid, which was washed with n-hexane (3 × 4 mL) and dried in vacuo. Yield: 280 mg (83%). Anal. Calcd. for C_40_H_58_N_4_ClRh: C, 65.52; H, 7.97; N, 7.64. Found: C, 65.63; H, 7.99; N, 7.62. ^1^H NMR (300.1 MHz, toluene-*d*_8_, 243 K): δ 7.4-7.2 (6H, H_Ph-IPr_), 6.63 (s, 2H, =CHN_IPr_), 5.93 (s, 2H, =CHN_IMe_), 4.41 and 2.58 (both sept, *J*_H-H_ = 6.6, 4H, CHMe_IPr_), 3.46 (s, 6H, Me_IMe_), 2.97 (m, 2H, =CH_coe_), 1.8-0.8 (12H, coe), 1.66, 1.42, 1.14, and 1.01 (all d, *J*_H-H_ = 6.6, 24H, CHMe_IPr_). ^13^C{^1^H}-APT NMR (75.5 MHz, toluene-*d*_8_, 243 K): δ 194.9 (d, *J*_C-Rh_ = 41.2, Rh-C_IPr_), 191.4 (d, *J*_C-Rh_ = 43.8, Rh-C_IMe_), 148.5 and 146.3 (both s, C_q-IPr_), 137.8 (s, C_q_N), 129.3 (s, CH_p-IPr_), 124.3 and 122.4 (both s, CH_m-IPr_), 123.7 (s, =CHN_IPr_), 120.2 (s, =CHN_IMe_), 54.1 (d, *J*_C-Rh_ = 16.8, =CH_coe_), 37.2 (s, Me_IMe_), 28.9 and 28.7 (both s, CHMe_IPr_), 31.7, 30.9, and 26.8 (all s, CH_2-coe_), 27.0, 26.8, 23.2, and 22.4 (all s, CHMe_IPr_). Figure S7. 1H NMR spectrum of 3a in toluene-d8 at 243 K. Figure S8. 13C{1H}-APT NMR spectrum of 3a in toluene-d8 at 243 K. Figure S9. 1H-1H COSY NMR spectrum of 3a in toluene-d8 at 243 K. Figure S10. 1H-13C HSQC NMR spectrum of 3a in toluene-d8 at 243 K. Figure S11. 1H-13C HSQC NMR spectrum of 3a in toluene-d8 at 243 K.

**Preparation ofRhCl)(η^2^-coe)(ICy)(IPr) (3b)** A solution of dinuclear complex **1a** (300 mg, 0.23 mmol) in 50 mL of toluene was treated with a solution of ICy (118 mg, 0.51 mmoles) in THF and stirred for 1h at room temperature. After filtration through Celite, the solvent was evaporated to dryness. The addition of n-hexane induced the precipitation of a yellow solid, which was washed with n-hexane (3 × 4 mL) and dried in vacuo. Yield: 387 mg (87%). Anal. Calcd. for C_50_H_74_N_4_ClRh: C, 69.07; H, 8.58; N, 6.44. Found: C, 68.83; H, 8.61; N, 6.62. ^1^H NMR (300.1 MHz, toluene-*d*_8_, 243 K): δ 7.4-7.2 (6H, H_Ph-IPr_), 6.52 (s, 2H, =CHN_IPr_), 6.34 (s, 2H, =CHN_ICy_), 5.10 (t, *J*_H-H_ = 10.1, 2H, CH_cy_), 4.15 and 2.70 (both sept, *J*_H-H_ = 6.1, 4H, CHMe_IPr_), 3.11 (m, 2H, =CH_coe_), 2.9-0.8 (32H, CH_2_), 1.66, 1.48, 1.12, and 1.00 (all d, *J*_H-H_ = 6.1, 24H, CHMe_IPr_). ^13^C{^1^H}-APT NMR (75.5 MHz, toluene-*d*_8_, 243 K): δ 192.8 (d, *J*_C-Rh_ = 41.2, Rh-C_IPr_), 188.0 (d, *J*_C-Rh_ = 45.5, Rh-C_ICy_), 148.0 and 146.0 (both s, C_q-IPr_), 138.2 (s, C_q_N), 129.2 (s, CH_p-IPr_), 124.9 and 122.5 (both s, CH_m-IPr_), 124.1 (s, =CHN_IPr_), 126.1 (s, =CHN_ICy_), 57.8 (s, CH_cy_), 54.5 (d, *J*_C-Rh_ = 16.4, =CH_coe_), 35.0, 33.4, 32.6, 30.8, 25.9, and 25.7 (all s, CH_2_), 28.8 and 28.7 (both s, CHMe_IPr_), 26.8, 26.7, 24.0, and 22.5 (all s, CHMe_IPr_). Figure S12. 1H NMR spectrum of 3b in toluene-d8 at 243 K. Figure S13. 13C{1H}-APT NMR spectrum of 3b in toluene-d8 at 243 K. Figure S14. 1H-1H COSY NMR spectrum of 3b in toluene-d8 at 243 K. Figure S15. 1H-13C HSQC NMR spectrum of 3b in toluene-d8 at 243 K. Figure S16. 1H-13C HMBC NMR spectrum of 3b in toluene-d8 at 243 K.

**Preparation ofRhCl(CO)(IMe)(IPr) (4a).** Carbon monoxide was bubbled through a yellow solution of **3a** (100 mg, 0.14 mmol) in 20 mL of toluene at room temperature for 30 min. After filtration through Celite, the solvent was evaporated to dryness. The addition of n-hexane induced the precipitation of a yellow solid, which was washed with n-hexane (3 × 4 mL) and dried in vacuo. Yield: 66 mg (71%). IR (cm^−1^, pure sample): 1935 ν(CO). HRMS (ESI) *m*/*z* Calcd for RhC_33_H_44_N_4_O (M-Cl): 615.2565. Found: 615.2568. ^1^H NMR (400.1 MHz, C_6_D_6_, 298 K): δ 7.46 (m, 2H, H_p-IPr_), 7.39 (m, 4H, H_m-IPr_), 6.90 (s, 2H, =CHN_IPr_), 5.91 (s, 2H, =CHN_IMe_), 3.59 (sept, *J*_H-H_ = 6.8, 4H, CHMe_IPr_), 3.30 (s, 6H, Me_IMe_), 1.71 and 1.26 (both d, *J*_H-H_ = 6.8, 24H, CHMe_IPr_). ^13^C{^1^H}-APT NMR (100.4 MHz, C_6_D_6_, 298 K): δ 193.2 (d, *J*_C-Rh_ = 44.7, Rh-C_IPr_), 188.5 (d, *J*_C-Rh_ = 83.0, CO), 185.3 (d, *J*_C-Rh_ = 42.7, Rh-C_IMe_), 147.0 (s, C_q-IPr_), 137.3 (s, C_q_N), 129.4 (s, CH_p-IPr_), 123.6 (s, =CHN_IPr_), 123.5 (s, CH_m-IPr_), 120.4 (s, =CHN_IMe_), 37.2 (s, Me_IMe_), 28.9 (s, CHMe_IPr_), 26.3 and 22.9 (both s, CHMe_IPr_). Figure S17. 1H NMR spectrum of 4a in C6D6 at 298 K. Figure S18. 13C{1H}-APT NMR spectrum of 4a in C6D6 at 298 K. Figure S19. 1H-1H COSY NMR spectrum of 4a in C6D6 at 298 K. Figure S20. 1H-13C HSQC NMR spectrum of 4a in C6D6 at 298 K. Figure S21. 1H-13C HMBC NMR spectrum of 4a in C6D6 at 298 K.

**Preparation ofRhCl(CO)(ICy)(IPr) (4b).** Carbon monoxide was bubbled through a yellow solution of **3b** (100 mg, 0.13 mmol) in 20 mL of toluene at room temperature for 30 min. After filtration through Celite, the solvent was evaporated to dryness. The addition of n-hexane induced the precipitation of a yellow solid, which was washed with n-hexane (3 × 4 mL) and dried in vacuo. Yield (76 mg, 83%). IR (cm^−1^, pure sample): 1937 ν(CO). HRMS (ESI) *m*/*z* Calcd for RhC_43_H_60_N_4_O (M-Cl): 751.3817. Found: 751.3795. ^1^H NMR (400.1 MHz, toluene-*d*_8_, 243 K): δ 7.32 (m, 2H, H_p-IPr_), 7.24 (m, 4H, H_m-IPr_), 6.76 (s, 2H, =CHN_IPr_), 6.34 (s, 2H, =CHN_ICy_), 4.84 (m, 2H, CH_cy_), 3.38 (sept, *J*_H-H_ = 6.5, 4H, CHMe_IPr_), 2.0–0.8 (all m, 20H, CH_2_), 1.52 and 1.13 (both d, *J*_H-H_ = 6.5, 24H, CHMe_IPr_). ^13^C{^1^H}-APT NMR (100.4 MHz, toluene-*d*_8_, 243 K): δ 193.8 (d, *J*_C-Rh_ = 41.6, Rh-C_IPr_), 188.6 (d, *J*_C-Rh_ = 81.4, CO), 183.6 (d, *J*_C-Rh_ = 40.6, Rh-C_ICy_), 147.3 (s, C_q-IPr_), 137.7 (s, C_q_N), 129.5 (s, CH_p-IPr_), 123.9 (s, CH_m-IPr_), 123.8 (s, =CHN_IPr_), 116.2 (s, =CHN_ICy_), 59.4 (s, CH_cy_), 33.9, 33.4, 25.8, 25.7, and 25.5 (all s, CH_2_), 28.9 (s, CHMe_IPr_), 26.5 and 23.1 (both s, CHMe_IPr_). Figure S22. 1H NMR spectrum of 4b in toluene-d8 at 243 K. Figure S23. 13C{1H}-APT NMR spectrum of 4b in toluene-d8 at 243 K. Figure S24. 1H-1H COSY NMR spectrum of 4b in toluene-d8 at 243 K. Figure S25. 1H-13C HSQC NMR spectrum of 4b in toluene-d8 at 243 K. Figure S26. 1H-13C HMBC NMR spectrum of 4b in toluene-d8 at 243 K.

**Figure 5:**
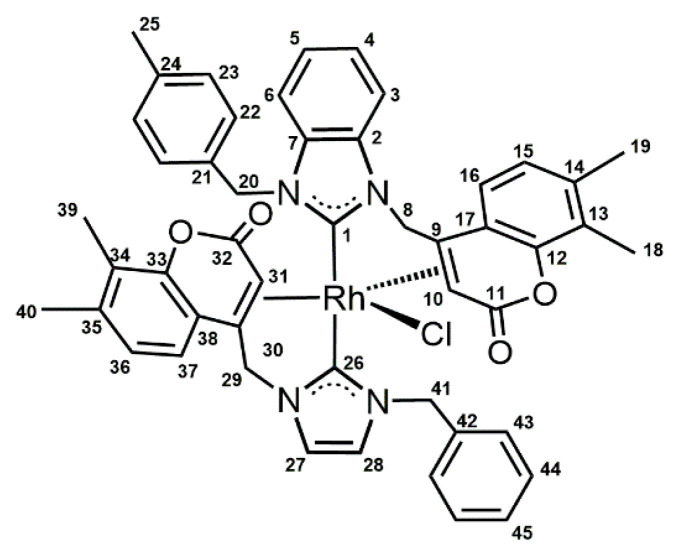
Numbering of atoms for complex **6**.

**Preparation ofRhCl(κ*C*,η^2^-BzICou^tol^)(κ*C*,η^2^-ICou^Bz^) (6).** (Figure 5) A mixture of RhCl(BzICou^tol^)(η^2^,η^2^-cod) (**5a**) (100 mg, 0.15 mmol), the imidazolium precursor [HICou^Bz^]Cl (60 mg, 0.15 mmol) and NaOCH_3_ (8 mg, 0.15 mmol) in 20 mL of THF was heated under reflux for 24 h. After this period, the mixture was cooled to 0 °C which resulted in the formation of a precipitate. The mother liquor was removed and the solid washed with cold THF (2 × 5 mL). Then, the solid was dissolved in 20 mL of CH_2_Cl_2_ and filtered through Celite. The solution was concentrated to ca. 1 mL, and then n-hexane was added to induce the precipitation of a white solid which was washed with n-hexane (3 × 5 mL) and dried in vacuo. Yield: 30 mg (45%). HRMS (ESI) *m*/*z* Calcd for RhC_49_H_44_N_4_O_4_ (M-Cl): 855.2412. Found: 855.2427. ^1^H NMR (400.1 MHz, CDCl_3_, 253 K): δ 7.68 (d, *J*_H-H_ = 8.0, 1H, H_16_), 7.54 (d, *J*_H-H_ = 7.9, 1H, H_37_), 7.53 (d, *J*_H-H_ = 7.8, 2H, H_43_), 7.30 (dd, *J*_H-H_ = 7.8, 7.4, 2H, H_44_), 7.29–7.22 (3H, H_22,45_), 7.14 and 5.20 (both d, *J*_H-H_ = 14.8, 2H, H_20_), 7.10–7.03 (6H, H_4,5,6,15,23_), 6.94 (both d, *J*_H-H_ = 7.7, 1H, H_36_), 6.82 (d, *J*_H-H_ = 7.2, 1H, H_3_), 6.79 and 4.99 (both d, *J*_H-H_ = 13.8, 2H, H_41_), 6.45 (d, *J*_H-H_ = 1.7, 1H, H_27_), 6.40 (d, *J*_H-H_ = 1.7, 1H, H_28_), 4.30 (br, 1H, H_10_), 4.19 and 3.55 (both d, *J*_H-H_ = 13.2, 2H, H_29_), 4.19 and 3.76 (both d, *J*_H-H_ = 13.2, 2H, H_8_), 4.15 (br, 1H, H_31_), 2.24 (s, 3H, H_19_), 2.23 (s, 3H, H_25_), 2.10 (s, 3H, H_18_), 1.80 (s, 3H, H_40_), 1.34 (s, 3H, H_39_). ^13^C{^1^H}-APT NMR (100.4 MHz, CDCl_3_, 253 K): δ 184.8 (d, *J*_C-Rh_ = 32.0, Rh-C_1_), 172.4 (d, *J*_C-Rh_ = 34.4, Rh-C_26_), 169.1 (s, O=C_11_), 169.0 (s, O=C_32_), 147.4 (s, C_12_), 146.7 (s, C_33_), 138.2 (s, C_35_), 138.1 (s, C_14_), 137.1 (s, C_24_), 137.0 (s, C_42_), 134.4 (s, C_7_), 133.5 (s, C_21_), 132.0 (s, C_2_), 129.8 (s, C_43_), 129.2 (s, C_23_), 128.6 (s, C_22_), 128.4 (s, C_44_), 128.0 (s, C_45_), 124.9 (s, C_13_), 124.5 (s, C_34_), 124.4 (s, C_15_), 124.1 (s, C_36_), 123.0 (s, C_4_), 122.9 (s, C_5_), 121.8 (s, C_28_), 121.1 (s, C_17_), 120.8 (s, C_38_), 119.2 (s, C_16_), 118.8 (s, C_37_), 117.9 (s, C_27_), 112.4 (s, C_6_), 109.2 (s, C_3_), 74.0 (d, *J*_C-Rh_ = 11.7, C_30_), 72.0 (d, *J*_C-Rh_ = 12.1, C_9_), 52.9 (s, C_41_), 52.5 (d, *J*_C-Rh_ = 7.6, C_31_), 51.6 (s, C_20_), 50.8 (d, *J*_C-Rh_ = 7.2, C_10_), 49.3 (s, C_29_), 46.8 (s, C_8_), 21.4 (s, C_25_), 20.4 (s, C_19_), 19.9 (s, C_40_), 11.8 (s, C_18_), 10.8 (s, C_39_). Figure S27. 1H NMR spectrum of 6 in toluene-d8 at 253 K. Figure S28. 13C{1H}-APT NMR spectrum of 6 in toluene-d8 at 253 K. Figure S29. 1H-13C HSQC NMR spectrum of 6 in toluene-d8 at 253 K. Figure S30. 1H-13C HMBC NMR spectrum of 6 in toluene-d8 at 253 K.

**Figure 6:**
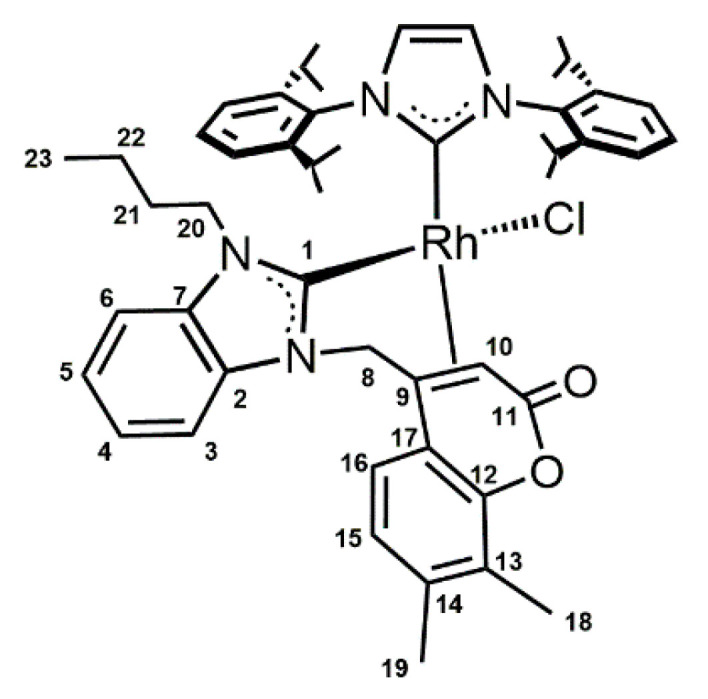
Numbering of atoms for complex **7**.

**Preparation ofRhCl(κ*C*,η^2^-BzICou^Bu^)(IPr) (7).** (Figure 6) A mixture of RhCl(BzICou^Bu^)*(*η^2^,η^2^-cod) (100 mg, 0.15 mmol) (**5b**) and free IPr (70, 0.18 mmol) in 20 mL of THF was heated under reflux for 24 h. After this period, THF was removed and then n-hexane was added to induce the precipitation of a white solid which was washed with n-hexane (3 × 5 mL) and dried in vacuo. Yield: 40 mg (29%). Anal. Calcd. for C_50_H_60_N_4_ClO_2_Rh: C, 67.67; H, 6.81; N, 6.31. Found: C, 67.62; H, 6.79; N, 6.42. ^1^H NMR (400.1 MHz, toluene-*d*_8_, 253 K): δ 7.37 and 7.24 (both t, *J*_H-H_ = 7.8, 2H, H_p-IPr_), 7.18 and 7.10 (both d, *J*_H-H_ = 7.8, 4H, H_m-IPr_), 6.90 and 6.87 (both dd, *J*_H-H_ = 7.4, 7.1, 2H, H_4,5_), 6.70 and 6.64 (both d, *J*_H-H_ = 7.5, 2H, H_3,6_), 6.65 (d, *J*_H-H_ = 8.0, 2H, H_15_), 6.46 and 6.39 (both d, *J*_H-H_ = 1.6, 2H, =CHN), 6.00 (d, *J*_H-H_ = 8.0, 1H, H_16_), 4.47, 3.26, 2.79, and 1.81 (all sept, *J*_H-H_ = 6.7, 4H, CHMe_IPr_), 4.16 and 2.41 (both d, *J*_H-H_ = 13.4, 2H, H_8_), 3.07 and 2.65 (both m, 2H, H_20_), 2.32 (s, 3H, H_18_), 2.20 (br, 1H, H_10_), 2.09 (s, 3H, H_19_), 1.35 and 1.28 (both m, 4H, H_21,22_), 1.01 (t, *J*_H-H_ = 7.3, 3H, H_23_), 1.74, 1.06, 0.95, 0.90, 0.87, 0.80, 0.53, and 0.53 (all d, *J*_H-H_ = 6.7, 24H, CHMe_IPr_). ^13^C{^1^H}-APT NMR (100.4 MHz, toluene-*d*_8_, 253 K): δ 196.1 (d, *J*_C-Rh_ = 52.2, Rh-C_BzICou_), 191.1 (d, *J*_C-Rh_ = 60.8, Rh-C_IPr_), 168.4 (s, O=C_11_), 150.6 (s, C_12_), 148.9, 146.9, 146.4, and 142.4 (all s, C_q-IPr_), 138.6 and 137.0 (both s, C_q_N), 136.5 (s, C_17_), 134.8 and 134.4 (both s, C_2,7_), 130.1 and 129.1 (both s, C_p-IPr_), 129.0, 128.4, 127.5, and 126.2 (all s, C_m-IPr_), 125.0 and 124.6 (both s, =CHN), 124.7 (s, C_15_), 124.2 (s, C_13_), 122.9 (s, C_14_), 122.1 and 121.7 (both s, C_4,5_), 121.4 (s, C_16_), 109.4 and 109.2 (both s, C_3,6_), 73.6 (d, *J*_C-Rh_ = 17.9, C_9_), 50.1, (s, C_8_), 46.2 (s, C_20_), 39.8 (d, *J*_C-Rh_ = 7.0, C_10_), 32.6 and 30.4 (both s, C_21,22_), 28.6, 28.2, 28.1, and 27.9 (all s, CHMe_IPr_), 26.9, 26.4, 25.8, 25.7, 25.6, 23.0, 22.8, and 20.4 (all s, CHMe_IPr_), 20.3 (s, C_19_), 14.2 (s, C_23_), 12.3 (s, C_18_). Figure S31. 1H NMR spectrum of 7 in toluene-d8 at 253 K. Figure S32. 13C{1H}-APT NMR spectrum of 7 in toluene-d8 at 253 K. Figure S33. 1H-13C HSQC NMR spectrum of 7 in toluene-d8 at 253 K. Figure S34. 1H-13C HMBC NMR spectrum of 7 in toluene-d8 at 253 K.

**Crystal Structure Determination**: Single crystals of **3a** and **7** for the X-ray diffraction studies were grown by slow diffusion of hexane into a saturated toluene solution (**3a**) or slow evaporation of CDCl_3_ solution in a NMR tube (**7**). X-ray diffraction data were collected at 100(2) K on a Bruker APEX DUO CCD diffractometer with graphite-monochromated Mo−Kα radiation (λ = 0.71073 Å) using ω rotations. The intensities were integrated and corrected for absorption effects with SAINT–PLUS [72] and SADABS [73] programs, both included in the APEX2 package. The structures were solved by the Patterson method with SHELXS-97 [74] and refined by full matrix least-squares on F^2^ with SHELXL-2014 [71], under WinGX [75]. CCDC 2204568 (**3a**) and 2204569 (7) contain the supplementary crystallographic data for this paper. These data can be obtained free of charge via http://www.ccdc.cam.ac.uk/conts/retrieving.html, accessed on 17 October 2022 (or from the CCDC, 12 Union Road, Cambridge CB2 1EZ, UK; Fax: +44-12-2333-6033; E-mail: deposit@ccdc.cam.ac.uk).

**Crystal data and structure refinement for 3a.** C_40_H_58_ClN_4_Rh, 733.26 g·mol^−1^, monoclinic, *P*2_1_/*n*, *a* = 11.2372(5) Å, *b* = 17.7258(7) Å, *c* = 19.8602(8) Å, *β* = 104.6600(10)°, *V* = 3827.1(3) Å^3^, *Z* = 4, *D*_calc_ = 1.273 g·cm^−3^, *μ* = 0.548 mm^−1^, *F*(000) = 1552, yellow prism, 0.160 × 0.080 × 0.050 mm^3^, *θ*_min_/*θ*_max_ 1.563/26.022°, index ranges –13 ≤ *h* ≤ 13, –21 ≤ *k* ≤ 21, –24 ≤*l* ≤ 24, reflections collected/independent 46716/7524 [R(int) = 0.0509], *T*_min_/*T*_max_ 0.9486/0.8307, data/restraints/parameters 7524/0/425, GooF(F^2^) = 1.015, *R*_1_ = 0.0319 [I > 2σ(I)], *wR*_2_ = 0.0744 (all data), largest diff. peak/hole 1.098/–0.473 e·Å^−3^.

**Crystal data and structure refinement for 7.** C_50_H_60_ClN_4_O_2_Rh·2.5 CH_2_Cl_2_, 1099.69 g·mol^−1^, monoclinic, *P*2_1_/*n*, *a* = 14.1094(12) Å, *b* = 20.7235(18) Å, *c* = 18.0797(16) Å, *β* = 96.0110(10)°, *V* = 5257.4(8) Å^3^, Z = 4, *D*_calc_ = 1.389 g·cm^–3^, *μ* = 0.673 mm^−1^, *F*(000) = 2284, red prism, 0.170 × 0.160 × 0.145 mm^3^, *θ*_min_/*θ*_max_ 1.965/26.372°, −17 ≤ *h* ≤ 17, −25 ≤ *k* ≤ 25, −22 ≤ *l* ≤ 22, reflections collected/independent 91967/10757 [R(int) = 0.0514], *T*_min_/*T*_max_ 0.8596/0.7205, data/restraints/parameters 10757/15/636, GooF(F^2^) = 1.118, *R*_1_ = 0.0445 [I > 2σ(I)], *wR*_2_ = 0.1104 (all data), largest diff. peak/hole 0.720/–1.106 e·Å^−3^.

## 4. Conclusions

Mixed bis-NHC rhodium(I) complexes of type RhCl(η^2^-olefin)(NHC)(NHC’) have been synthesized following a stepwise procedure. It has been revealed that the steric hindrance imparted by the wingtips of the carbene ligands as well as that of the η^2^-olefin is critical. Thus, bis-NHC derivatives containing both bulky IPr or IMes could be accessed only for smaller η^2^-ethylene, whereas the steric relief in IMe or ICy allows for the preparation of mixed bis-NHC complexes containing a η^2^-coe ligand. Regarding the use of RhCl(NHC)(η^2^,η^2^-cod) precursors, in contrast to the stability observed for IPr derivative, the coumarin-functionalized NHC ligands facilitates decoordination of the diolefin, enabling the straightforward introduction of a second NHC. Thus, the pentacoordinated mixed bis-NHC complexes bearing coumarin functionalities adopt a trans-NHC disposition, whereas the square planar IPr-BzIcou species presents an uncommon cis-NHC configuration, exhibiting an anagostic interaction between the metal atom and one IPr methyl group.

## Data Availability

Not available.

## Supplementary Materials

The following supporting information can be downloaded at: https://www.mdpi.com/article/10.3390/molecules27207002/s1, Miscellaneous information. Figure S1. Variable-temperature 1H NMR spectra of 3a in toluene-d8. Figure S2. 1H NMR spectrum of 2 in C6D6 at 298 K. Figure S3. 13C{1H}-APT NMR spectrum of 2 in C6D6 at 298 K. Figure S4. 1H-1H COSY NMR spectrum of 2 in C6D6 at 298 K. Figure S5. 1H-13C HSQC NMR spectrum of 2 in C6D6 at 298 K. Figure S6. 1H-13C HMBC NMR spectrum of 2 in C6D6 at 298 K. Figure S7. 1H NMR spectrum of 3a in toluene-d8 at 243 K. Figure S8. 13C{1H}-APT NMR spectrum of 3a in toluene-d8 at 243 K. Figure S9. 1H-1H COSY NMR spectrum of 3a in toluene-d8 at 243 K. Figure S10. 1H-13C HSQC NMR spectrum of 3a in toluene-d8 at 243 K. Figure S11. 1H-13C HSQC NMR spectrum of 3a in toluene-d8 at 243 K. Figure S12. 1H NMR spectrum of 3b in toluene-d8 at 243 K. Figure S13. 13C{1H}-APT NMR spectrum of 3b in toluene-d8 at 243 K. Figure S14. 1H-1H COSY NMR spectrum of 3b in toluene-d8 at 243 K. Figure S15. 1H-13C HSQC NMR spectrum of 3b in toluene-d8 at 243 K. Figure S16. 1H-13C HMBC NMR spectrum of 3b in toluene-d8 at 243 K. Figure S17. 1H NMR spectrum of 4a in C6D6 at 298 K. Figure S18. 13C{1H}-APT NMR spectrum of 4a in C6D6 at 298 K. Figure S19. 1H-1H COSY NMR spectrum of 4a in C6D6 at 298 K. Figure S20. 1H-13C HSQC NMR spectrum of 4a in C6D6 at 298 K. Figure S21. 1H-13C HMBC NMR spectrum of 4a in C6D6 at 298 K. Figure S22. 1H NMR spectrum of 4b in toluene-d8 at 243 K. Figure S23. 13C{1H}-APT NMR spectrum of 4b in toluene-d8 at 243 K. Figure S24. 1H-1H COSY NMR spectrum of 4b in toluene-d8 at 243 K. Figure S25. 1H-13C HSQC NMR spectrum of 4b in toluene-d8 at 243 K. Figure S26. 1H-13C HMBC NMR spectrum of 4b in toluene-d8 at 243 K. Figure S27. 1H NMR spectrum of 6 in toluene-d8 at 253 K. Figure S27. 1H NMR spectrum of 6 in toluene-d8 at 253 K. Figure S29. 1H-13C HSQC NMR spectrum of 6 in toluene-d8 at 253 K. Figure S30. 1H-13C HMBC NMR spectrum of 6 in toluene-d8 at 253 K. Figure S31. 1H NMR spectrum of 7 in toluene-d8 at 253 K. Figure S32. 13C{1H}-APT NMR spectrum of 7 in toluene-d8 at 253 K. Figure S33. 1H-13C HSQC NMR spectrum of 7 in toluene-d8 at 253 K. Figure S34. 1H-13C HSQC NMR spectrum of 7 in toluene-d8 at 253 K.

## Sample Availability

Samples of the compounds are available from the authors.
