# Supplementary material for: Preparation of Mixed Bis-N-Heterocyclic Carbene Rhodium(I) Complexes
Source: Molecules. 2022 Oct 18;27(20):7002. doi: 10.3390/molecules27207002 (PMC9611579; doi:10.3390/molecules27207002)
Supplement: Supplementary file 1 [file molecules-27-07002-s001.zip › molecules-1979363-supplementary.pdf]

Supporting Information

Preparation of Mixed Bis-N-Heterocyclic Carbene Rhodium(I) Complexes

Ramón Azpíroz[1,*], *Mert Olgun Karataş*[1,2], *Vincenzo Passarelli*[1], *Ismail Özdemir*[2], *Jesús J. Pérez-Torrente*[1], *Ricardo Castarlenas*[1,*]

1- Departamento de Química Inorgánica – Instituto de Síntesis Química y Catálisis Homogénea-ISQCH, Universidad de Zaragoza – CSIC. C/ Pedro Cerbuna 12, 50009 Zaragoza, Spain

2- Department of Chemistry, Faculty of Sciences, Inonu University, 44280, Malatya, Turkey

Table of contents

Variable-temperature ^{1}H NMR spectra of **3a** S2

NMR spectra of organometallic complexes S3

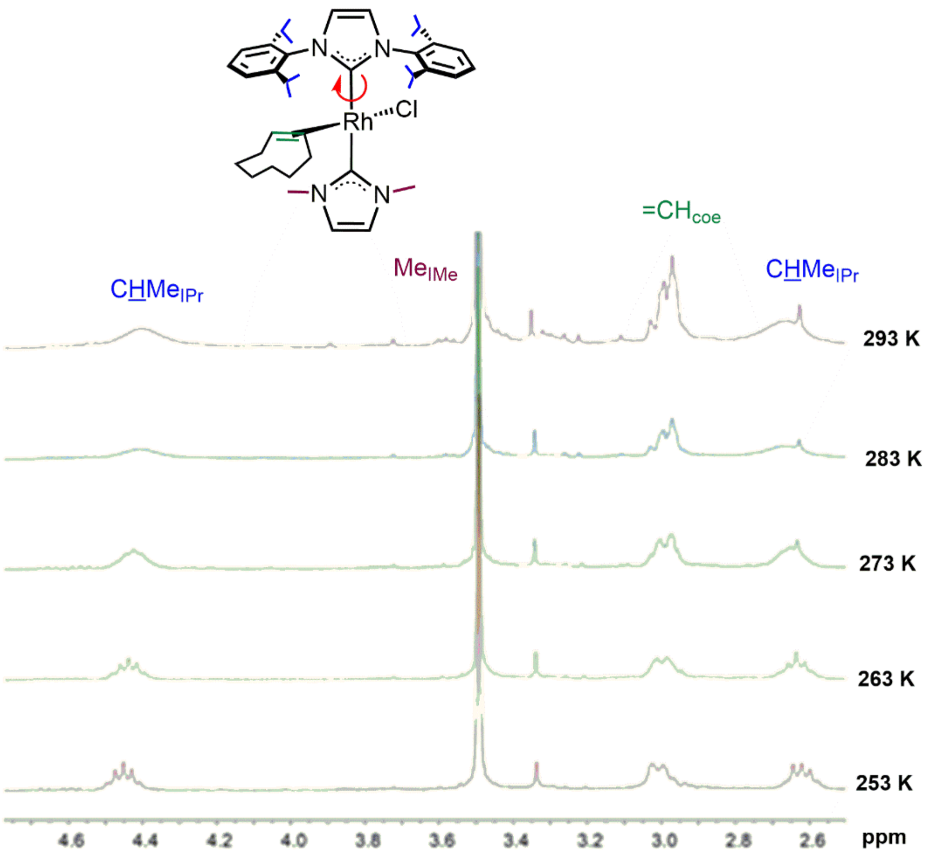

Figure S1. Variable-temperature ^{1}H NMR spectra of **3a** in toluene-d_8.

NMR spectra of Organometallic complexes

RhCl(η^2-CH_2=CH_2)(IPr)$_2$ (2)

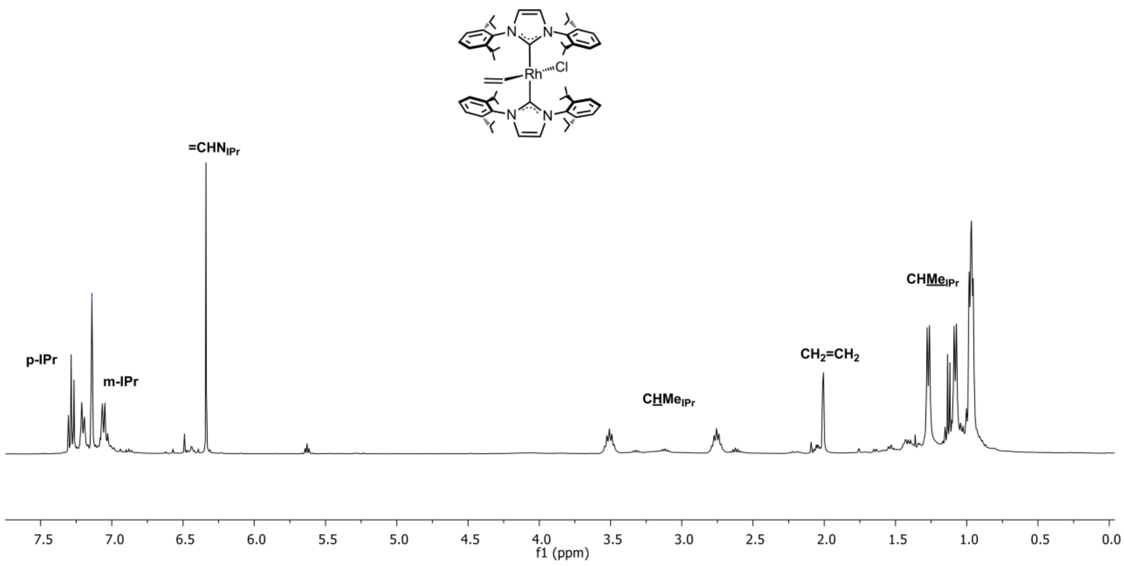

Figure S2. ^{1}H NMR spectrum of **2** in C_6D_6 at 298 K.

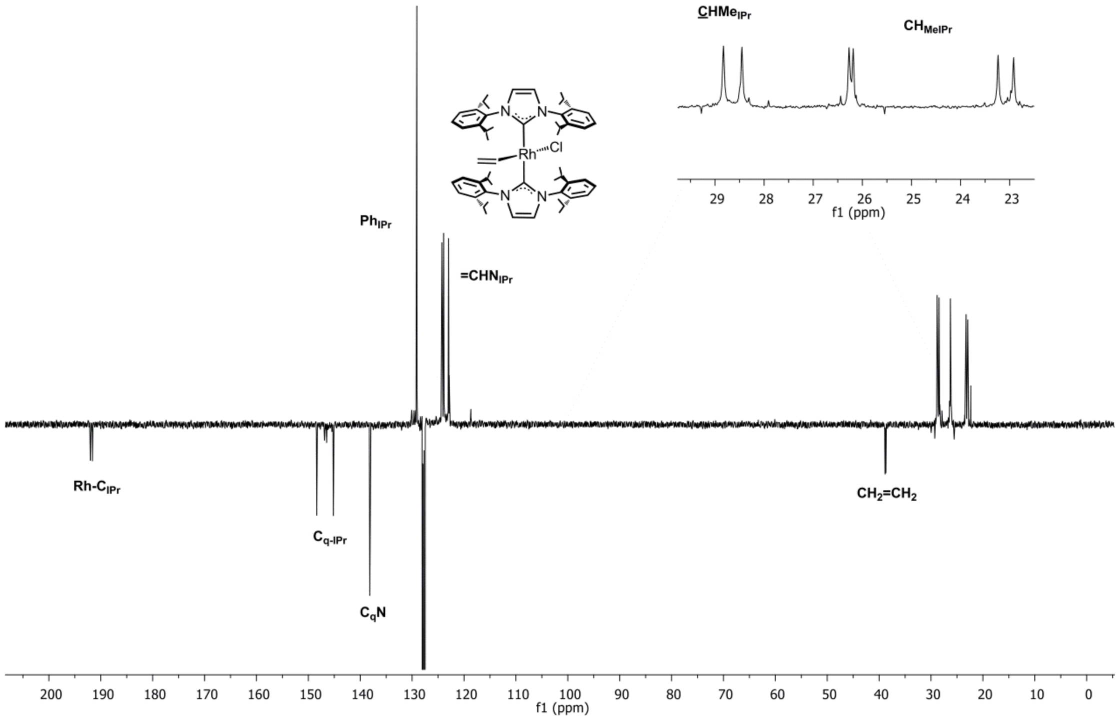

Figure S3. $^{13}C\{^1H\}$-APT NMR spectrum of **2** in C_6D_6 at 298 K.

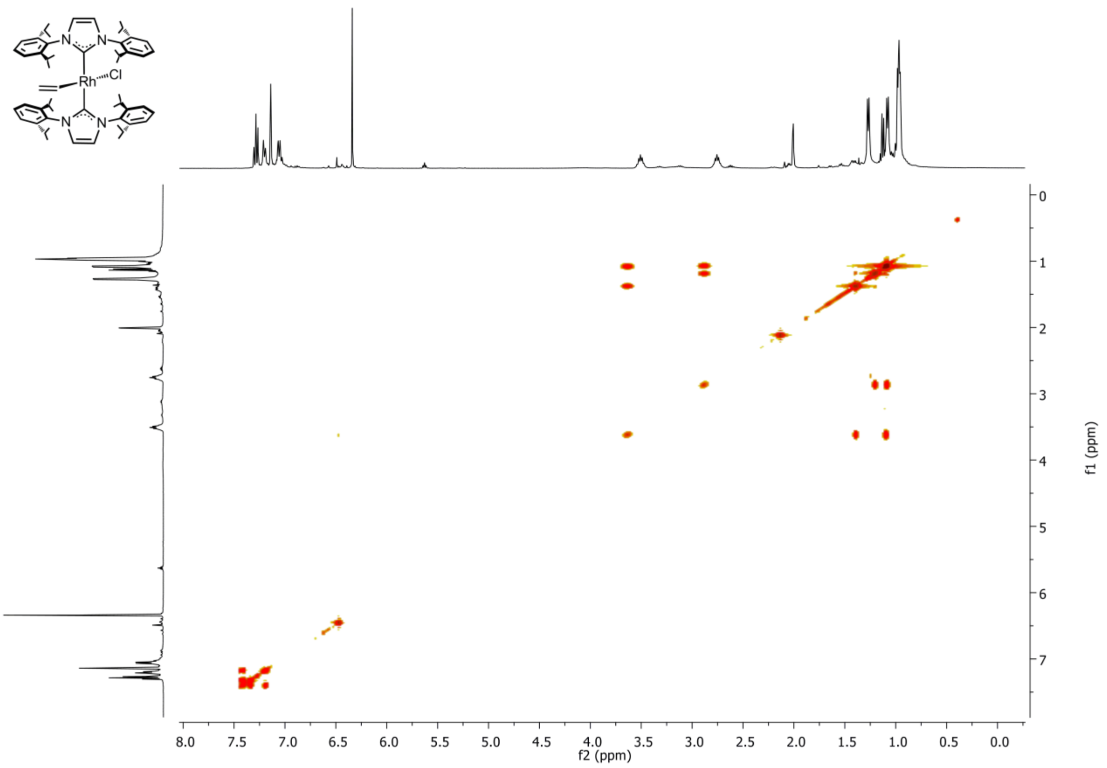

Figure S4. ^1H-^1H COSY NMR spectrum of **2** in C_6D_6 at 298 K.

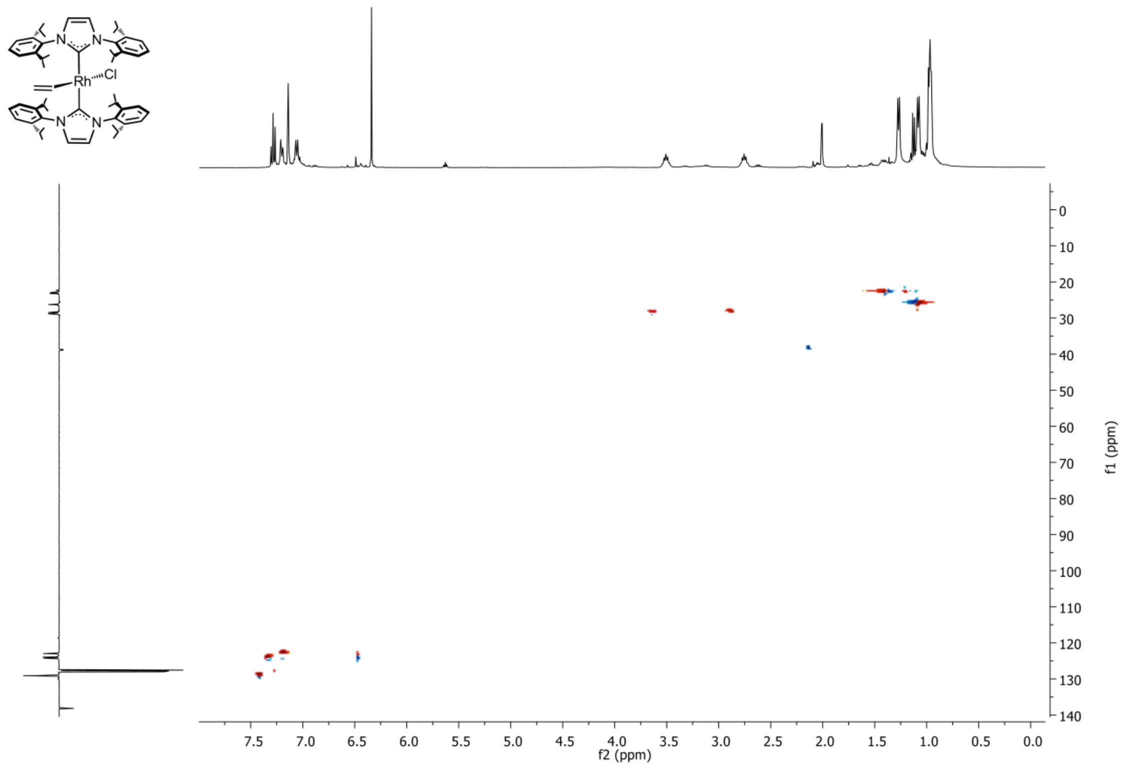

Figure S5. ^1H-^{13}C HSQC NMR spectrum of **2** in C_6D_6 at 298 K.

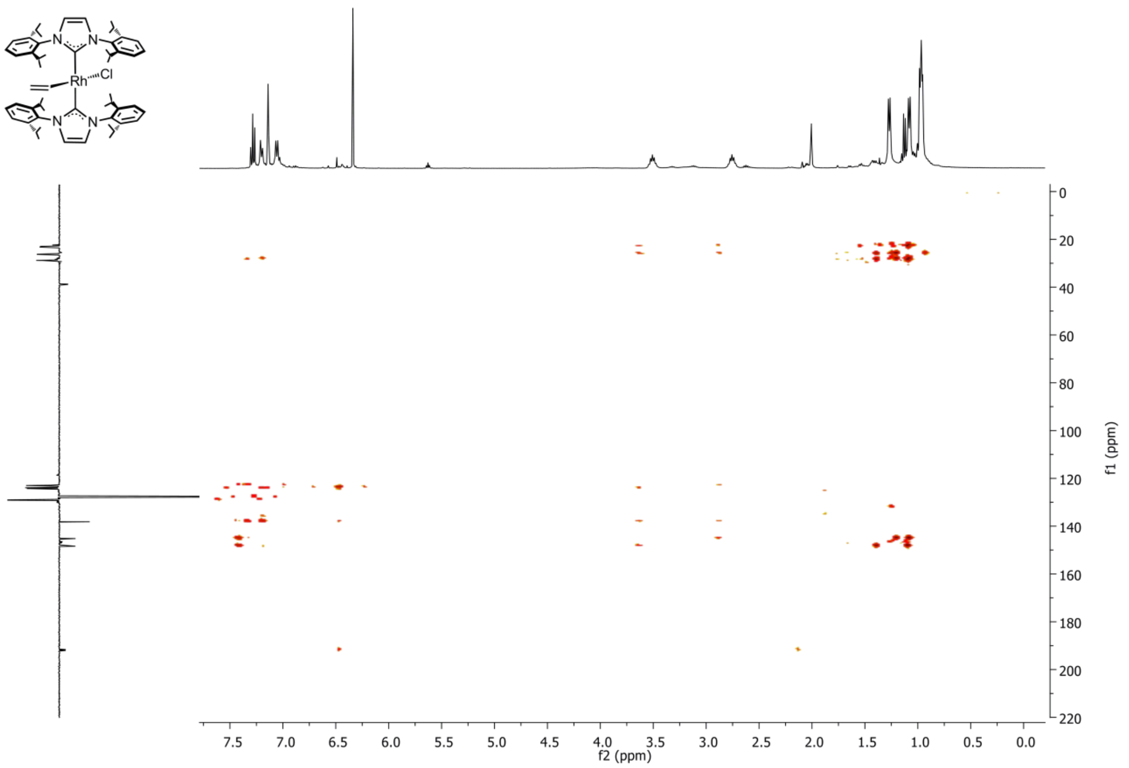

Figure S6. ^{1}H-^{13}C HMBC NMR spectrum of **2** in C_6D_6 at 298 K.

RhCl(η²-coe)(IMe)(IPr) (3a)

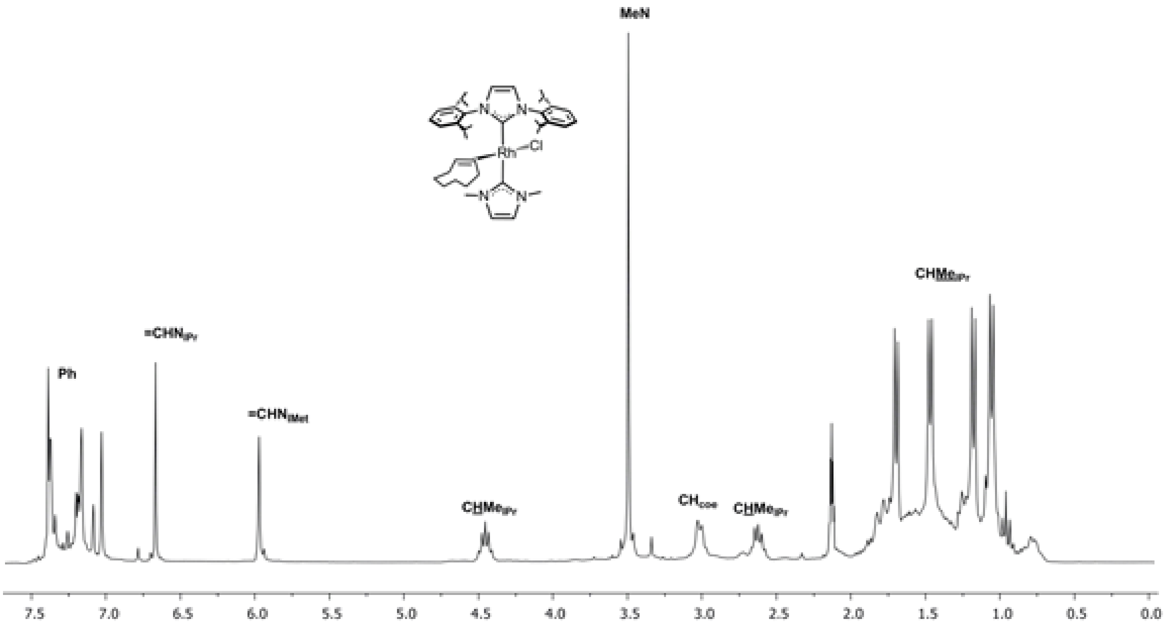

Figure S7. ^{1}H NMR spectrum of **3a** in toluene-d_8 at 243 K.

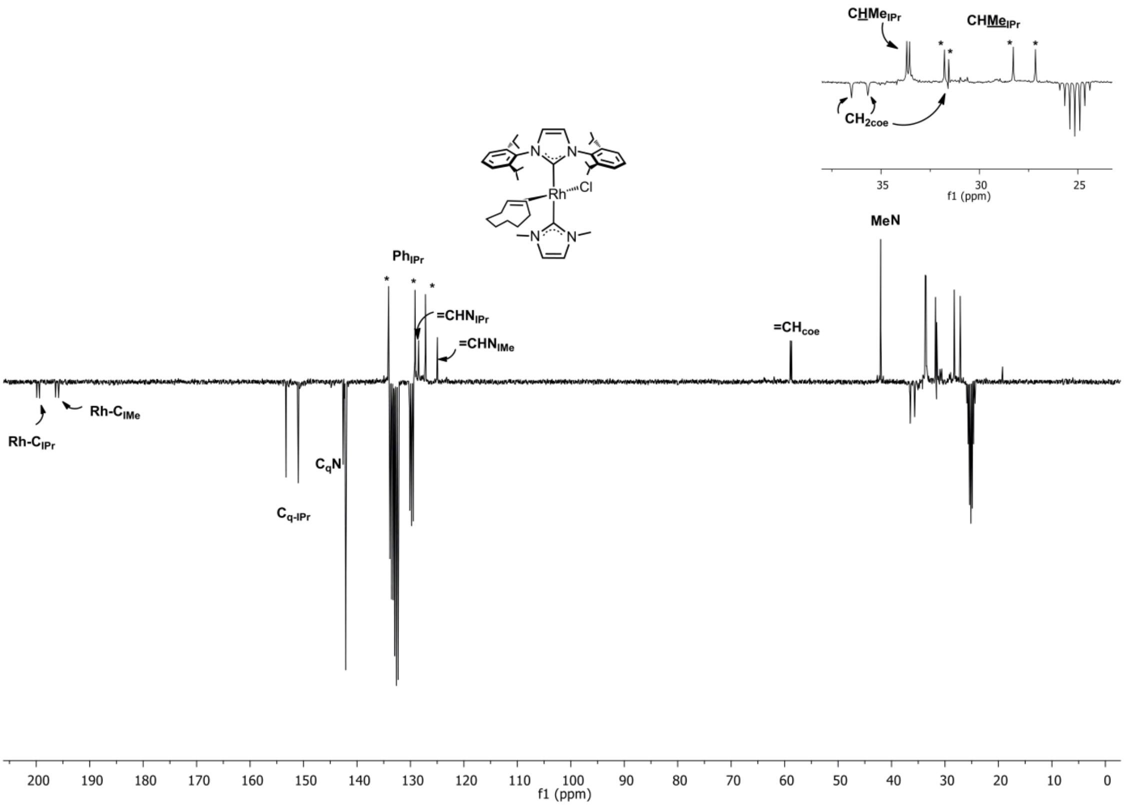

Figure S8. $^{13}C\{^{1}H\}$-APT NMR spectrum of **3a** in toluene-d_8 at 243 K.

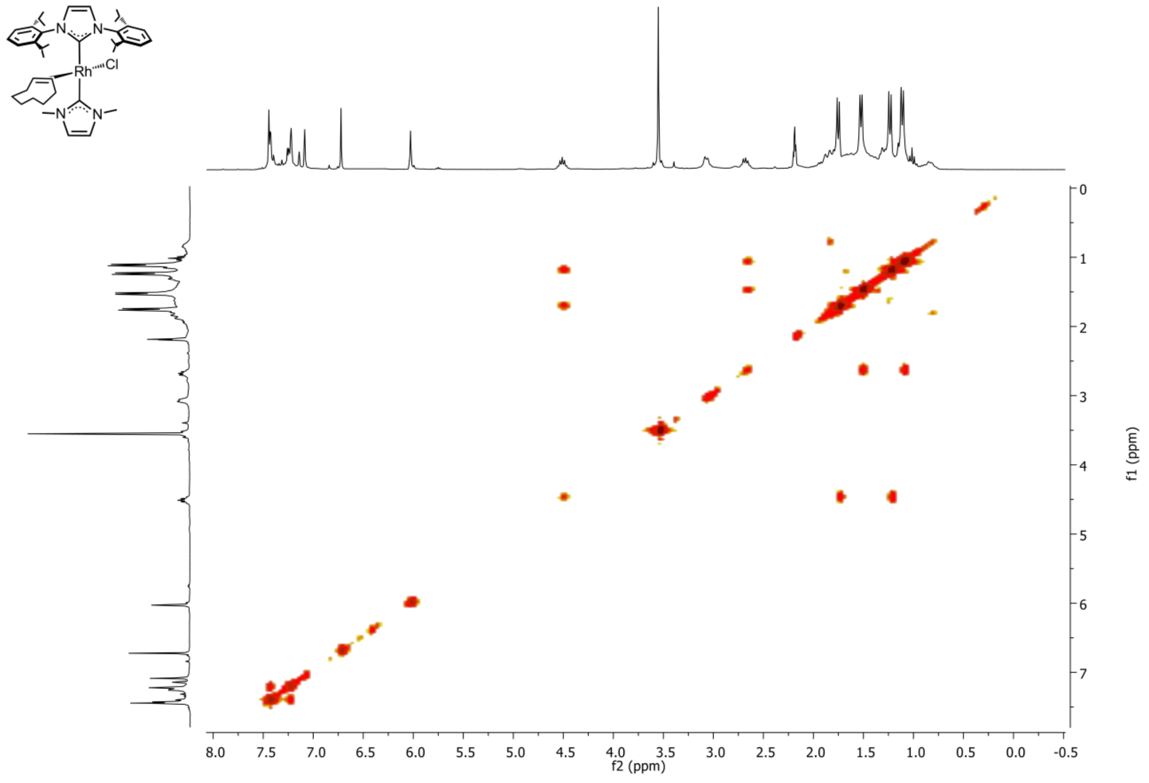

Figure S9. ^{1}H-^{1}H COSY NMR spectrum of **3a** in toluene-d_8 at 243 K.

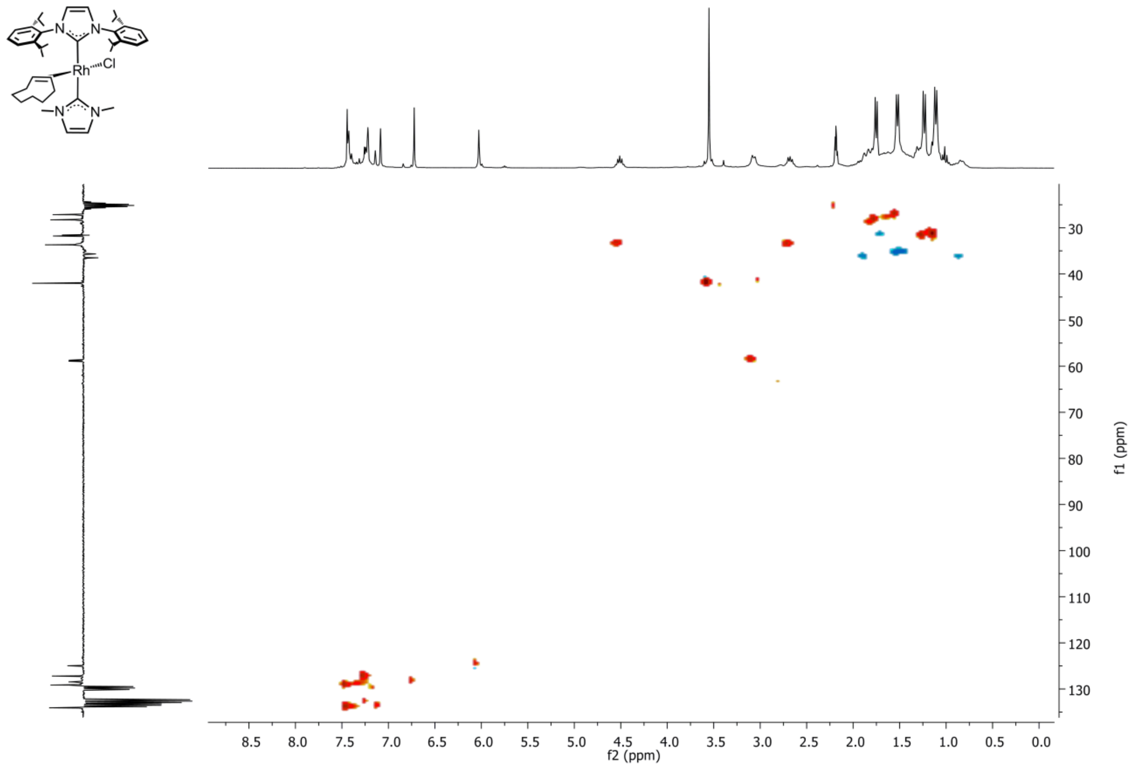

Figure S10. ^{1}H-^{13}C HSQC NMR spectrum of **3a** in toluene-d_8 at 243 K.

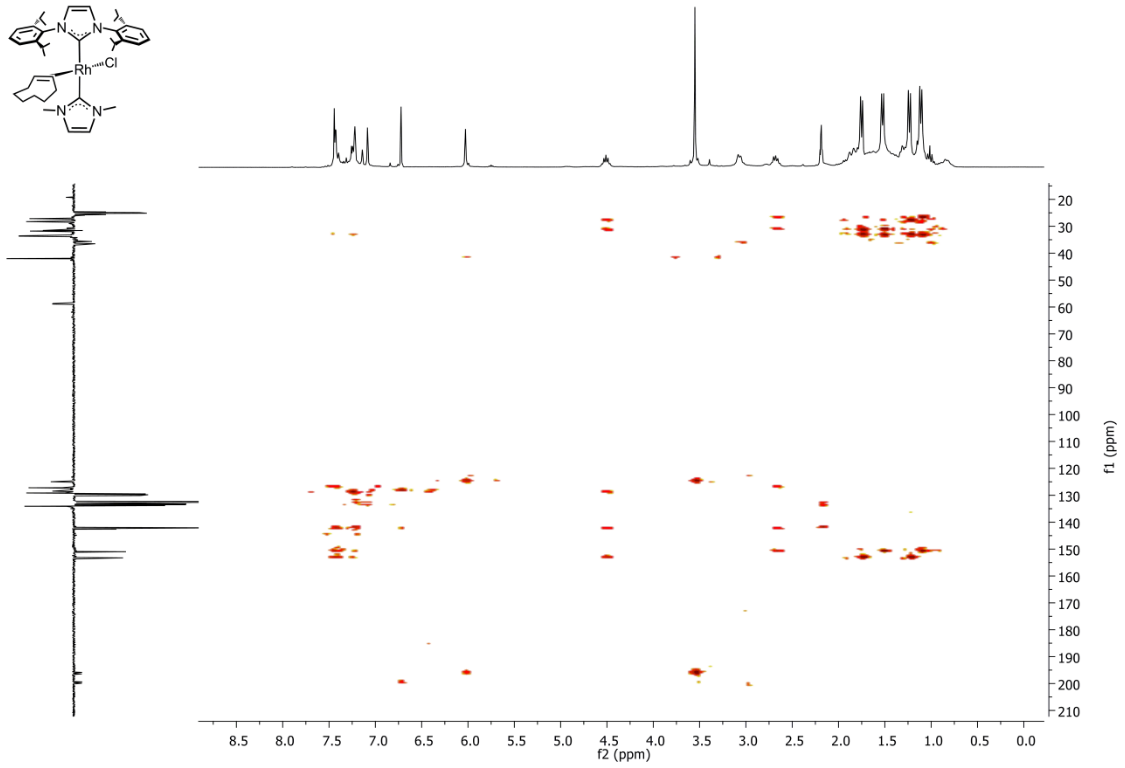

Figure S11. ^{1}H-^{13}C HSQC NMR spectrum of **3a** in toluene-d_8 at 243 K.

RhCl(η²-coe)(ICy)(IPr) (3b)

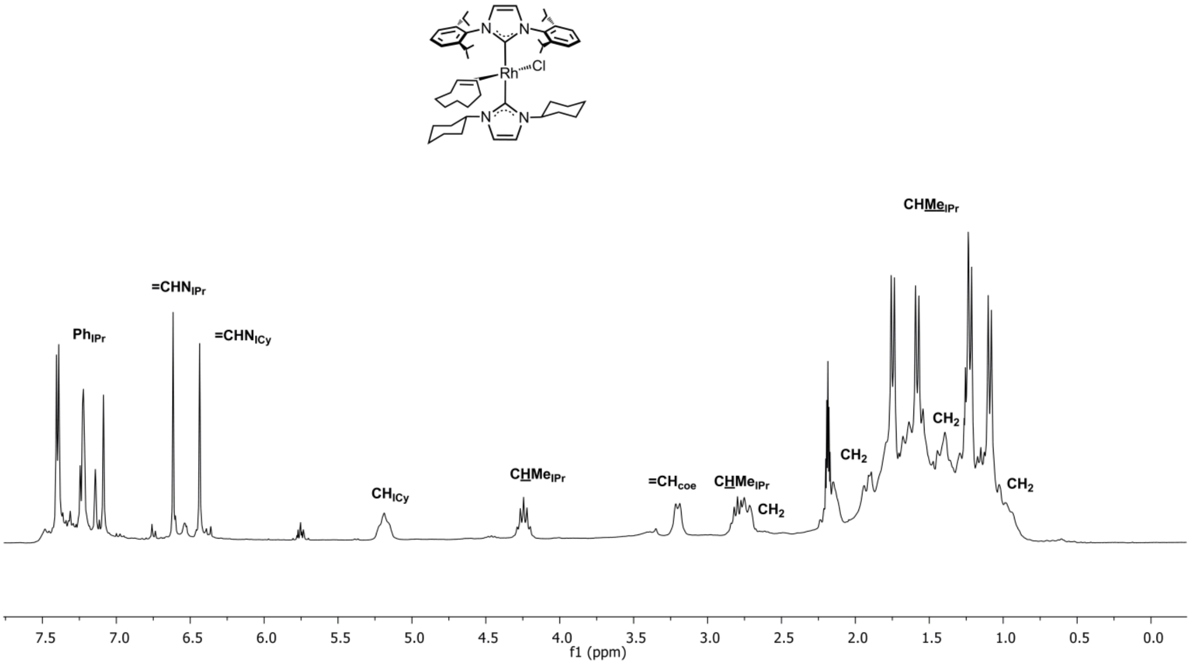

Figure S12. ^{1}H NMR spectrum of **3b** in toluene-d_8 at 243 K.

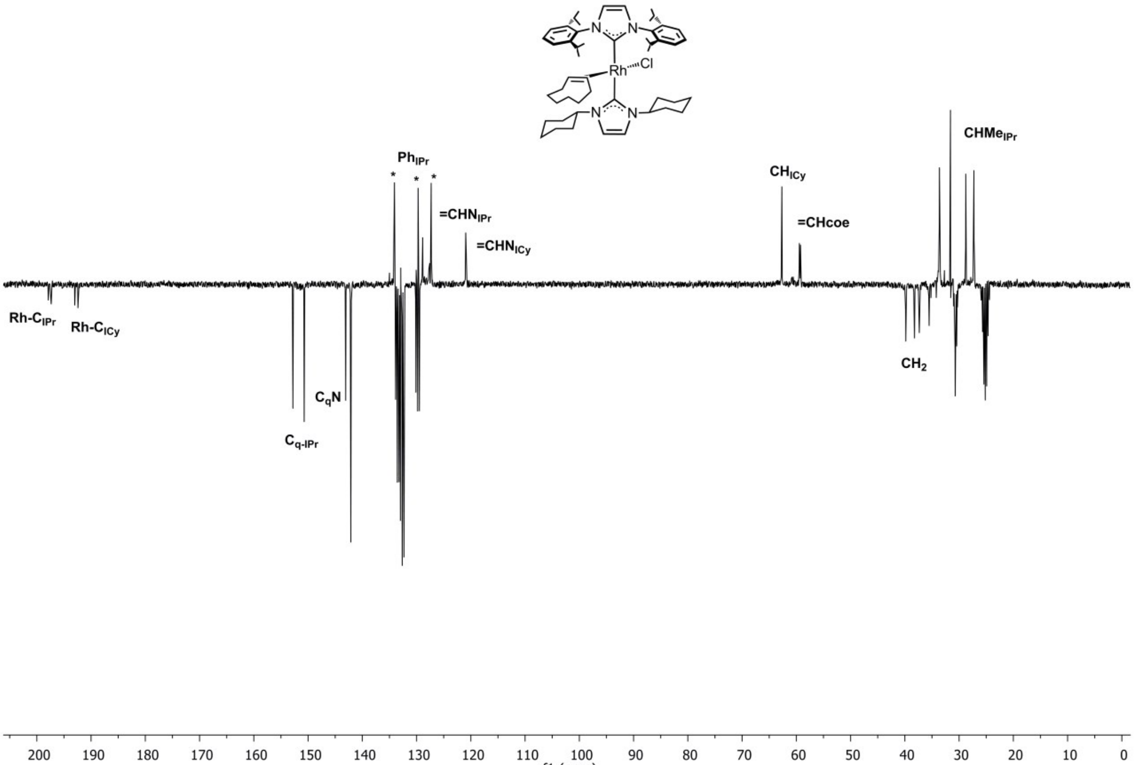

Figure S13. $^{13}C\{^{1}H\}$-APT NMR spectrum of **3b** in toluene-d_8 at 243 K.

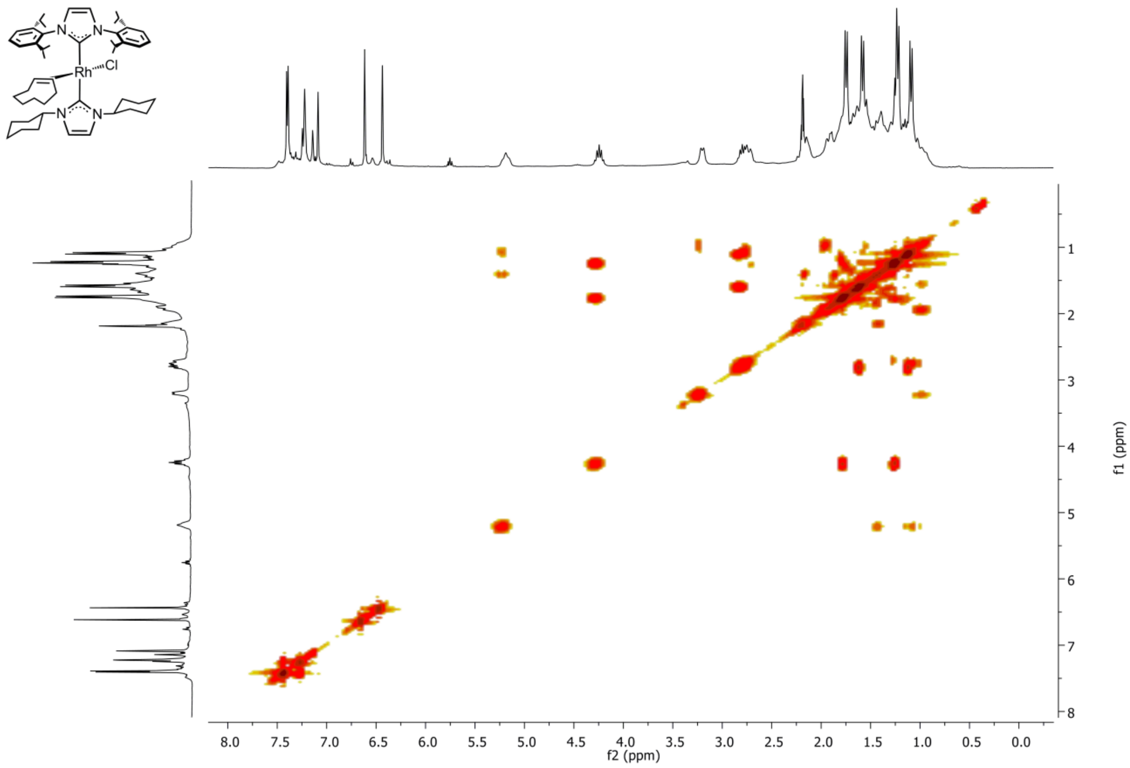

Figure S14. ^{1}H-^{1}H COSY NMR spectrum of **3b** in toluene-d_8 at 243 K.

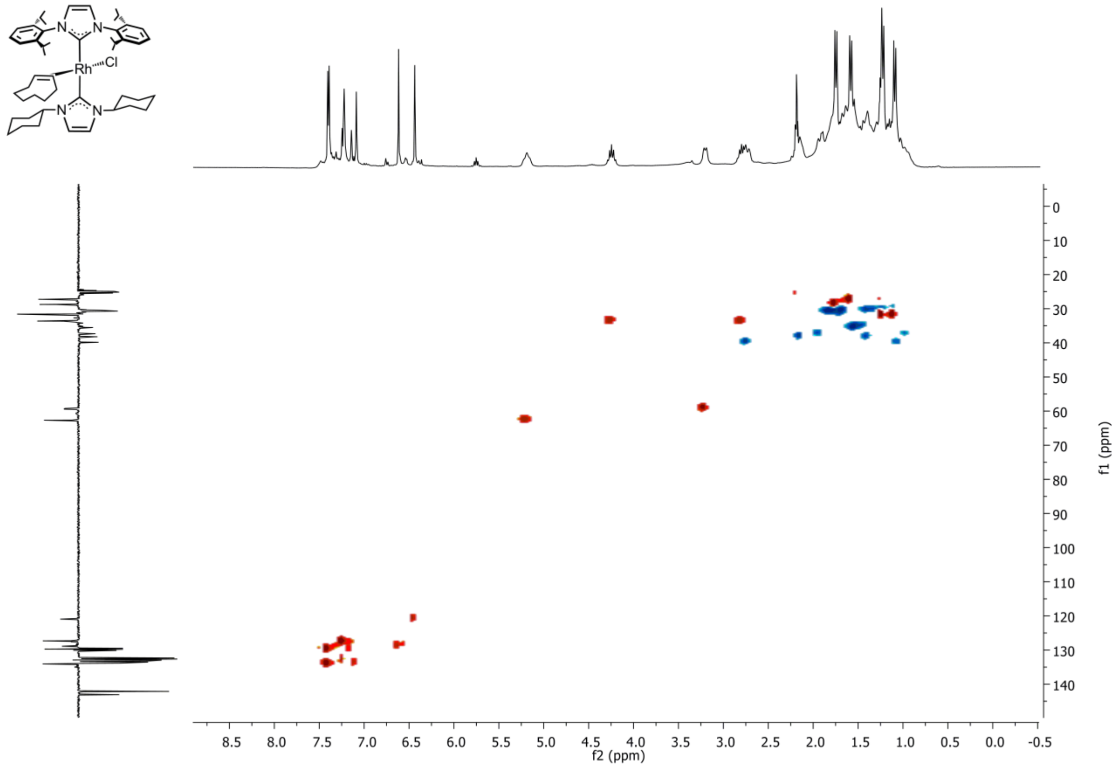

Figure S15. ^{1}H-^{13}C HSQC NMR spectrum of **3b** in toluene-d_8 at 243 K.

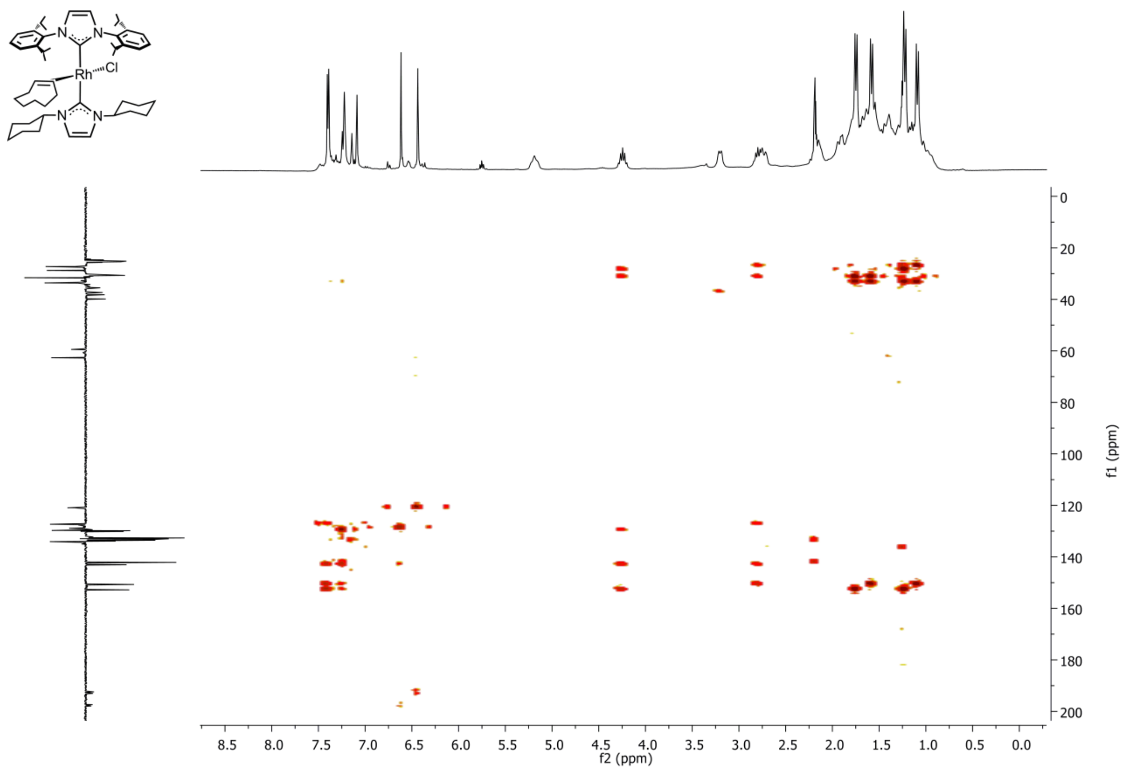

Figure S16. ^{1}H-^{13}C HMBC NMR spectrum of **3b** in toluene-d_8 at 243 K.

RhCl(CO)(IMe)(IPr) (4a)

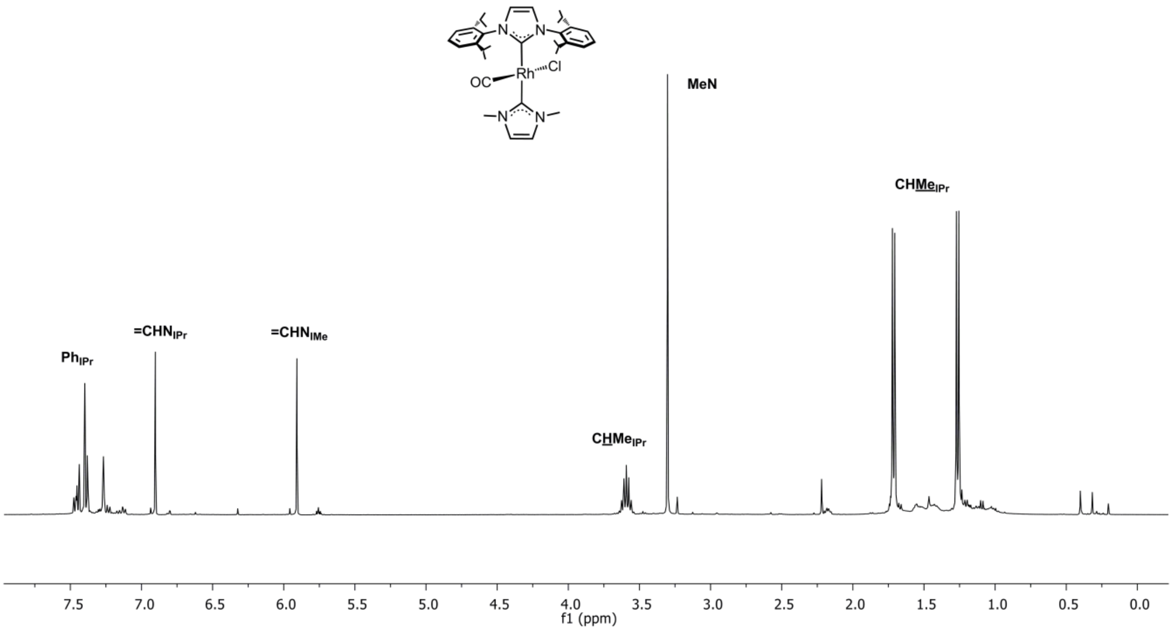

Figure S17. ^{1}H NMR spectrum of **4a** in C_6D_6 at 298 K.

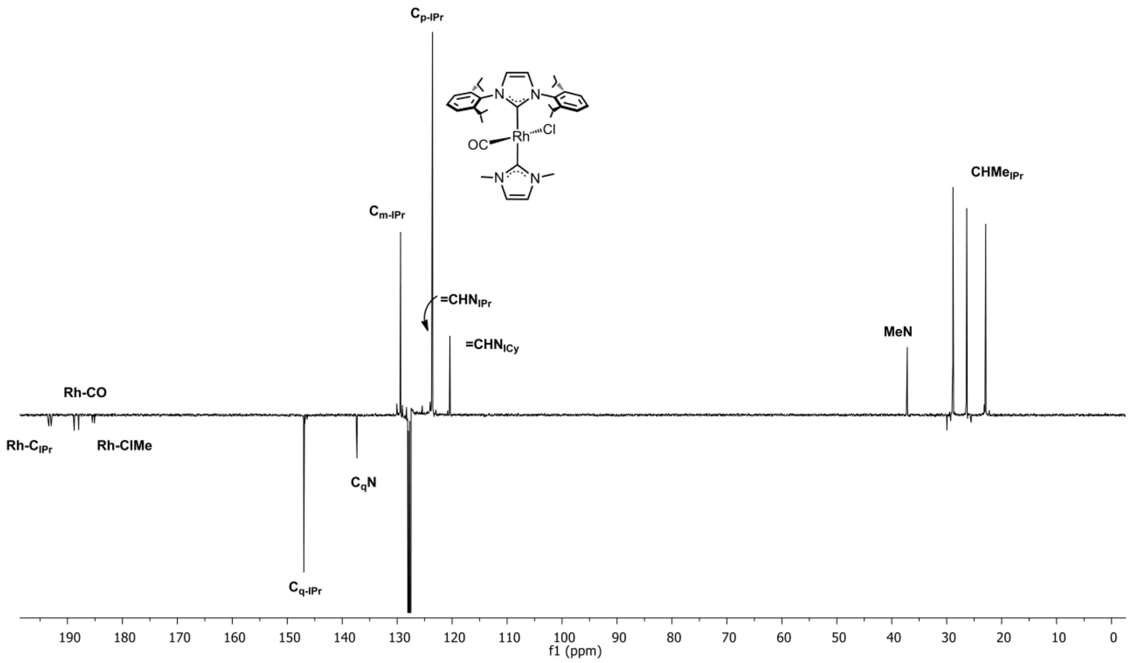

Figure S18. ${}^{13}C\{{}^{1}H\}$-APT NMR spectrum of **4a** in C_6D_6 at 298 K.

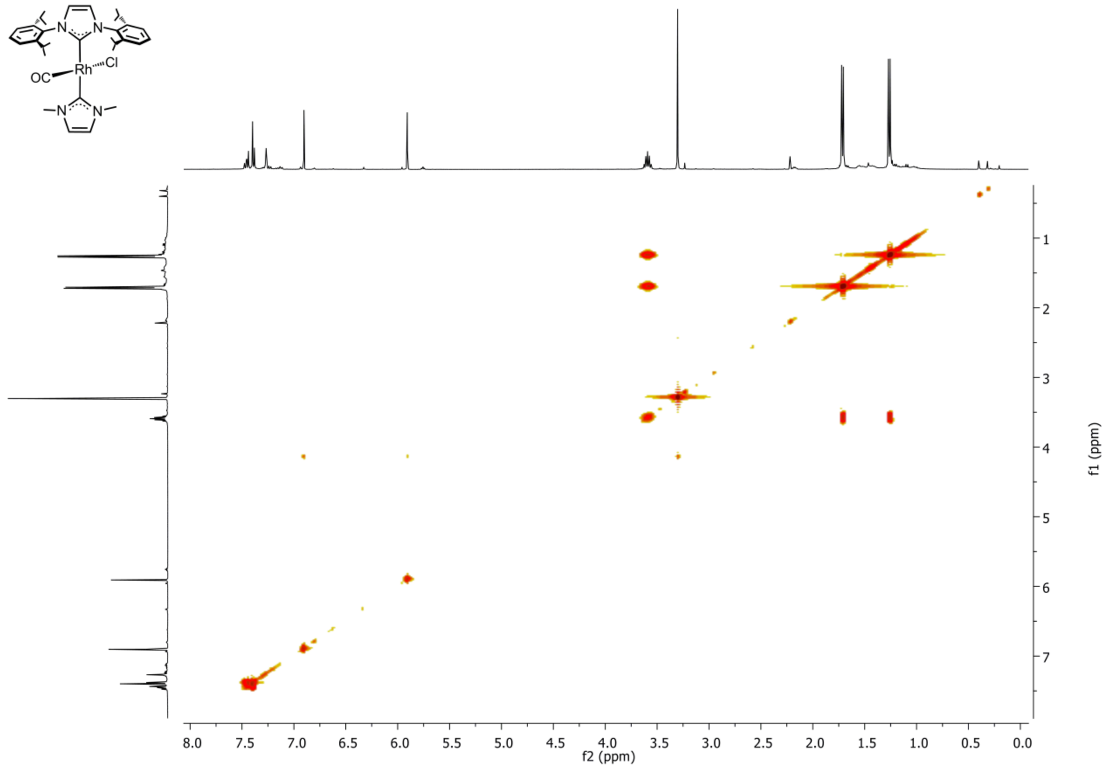

Figure S19. ${}^{1}H$-${}^{1}H$ COSY NMR spectrum of **4a** in C_6D_6 at 298 K.

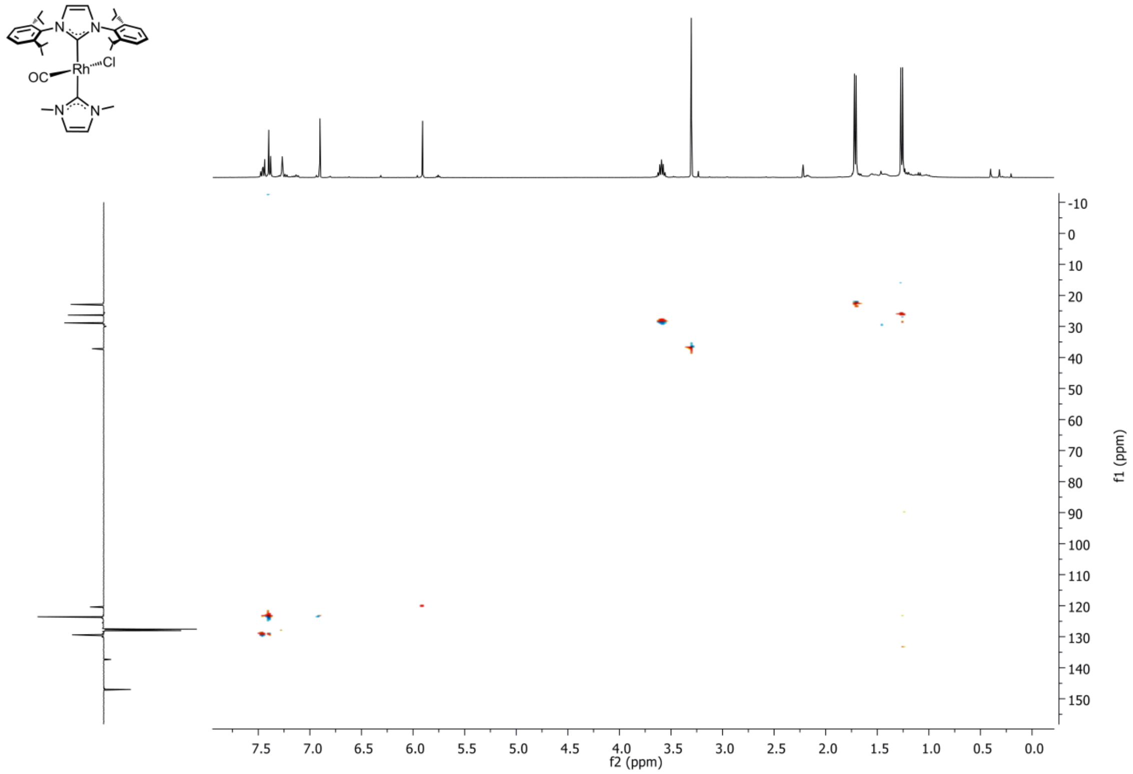

Figure S20. ^{1}H-^{13}C HSQC NMR spectrum of **4a** in C_6D_6 at 298 K.

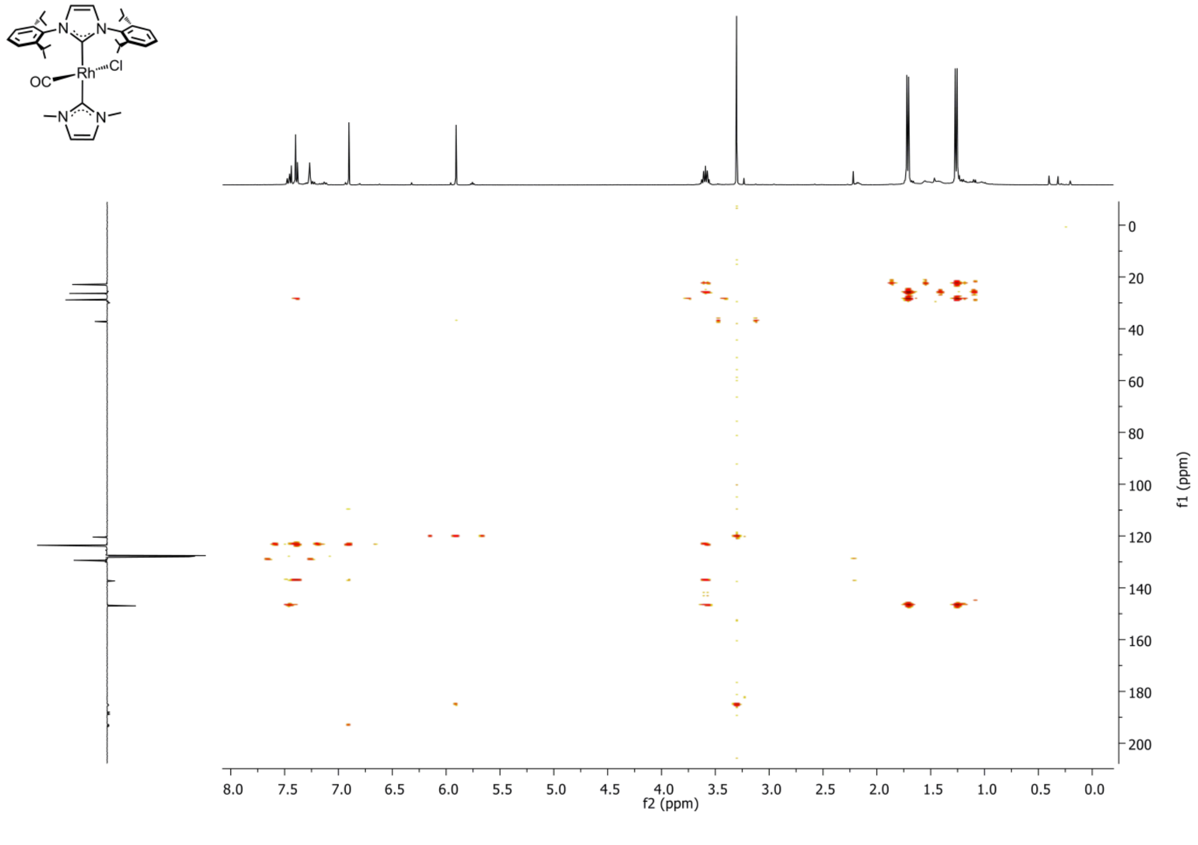

Figure S21. ^{1}H-^{13}C HMBC NMR spectrum of **4a** in C_6D_6 at 298 K.

RhCl(CO)(ICy)(IPr) (4b)

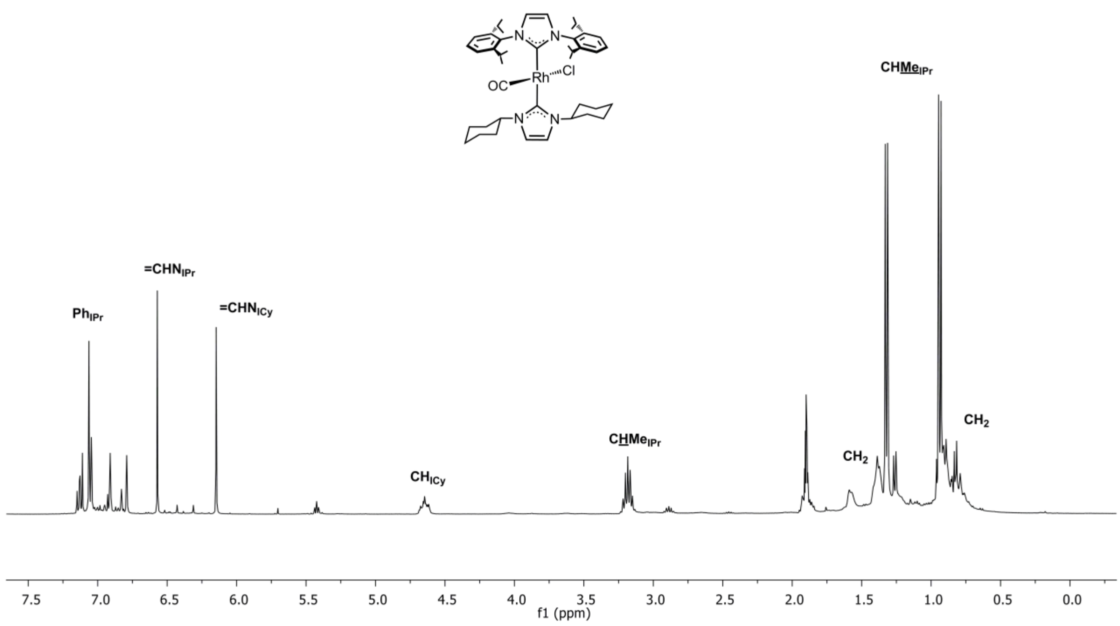

Figure S22. ^{1}H NMR spectrum of **4b** in toluene-d_8 at 243 K.

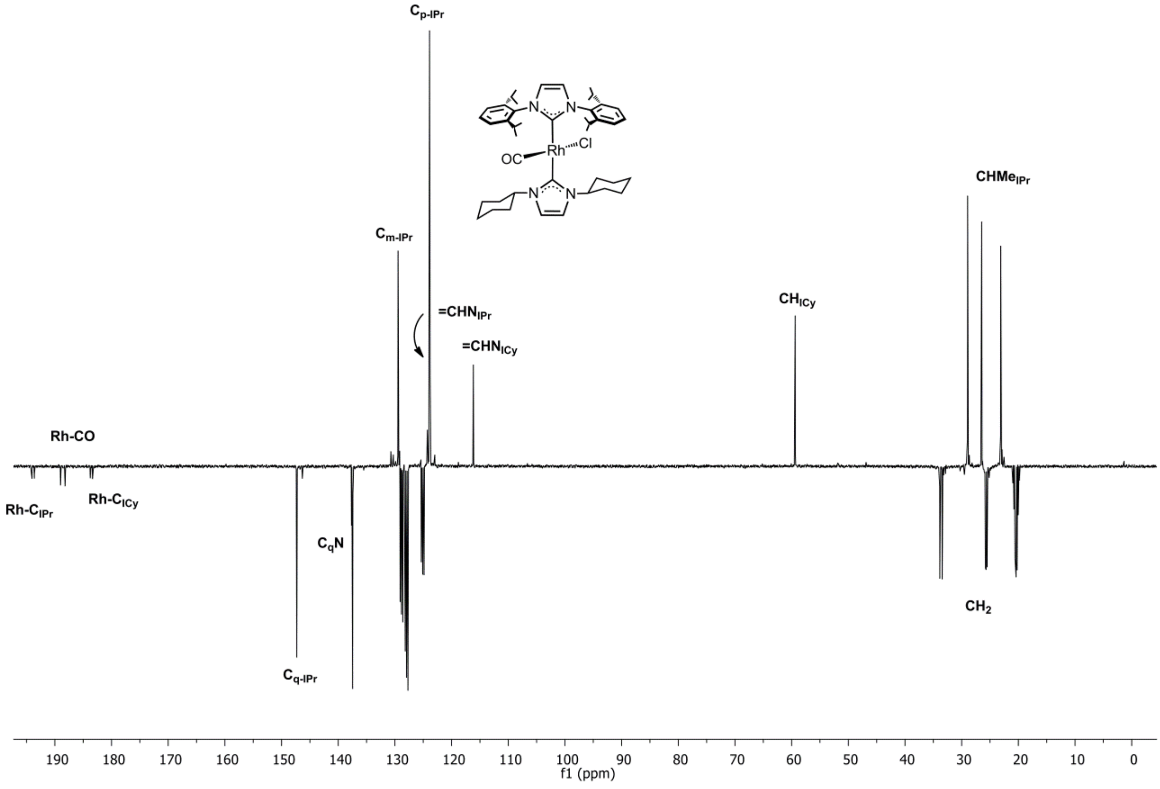

Figure S23. ^{13}C{^{1}H}-APT NMR spectrum of **4b** in toluene-d_8 at 243 K.

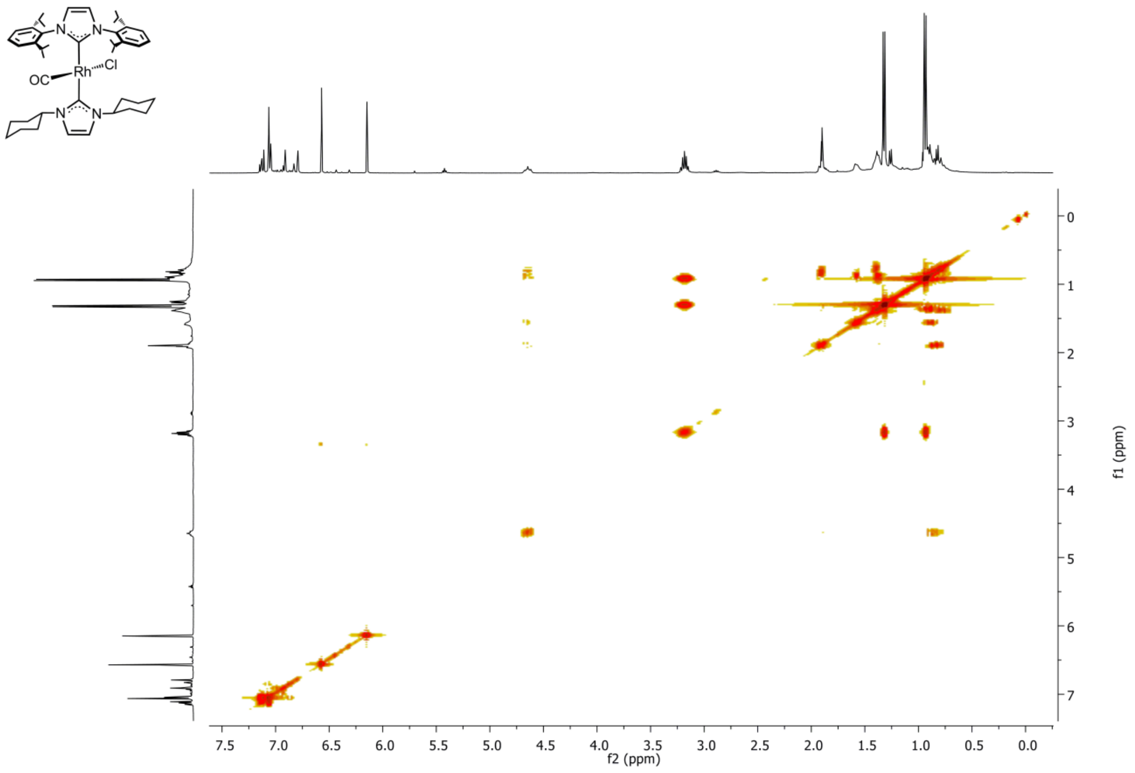

Figure S24. ^{1}H-^{1}H COSY NMR spectrum of **4b** in toluene-d_8 at 243 K.

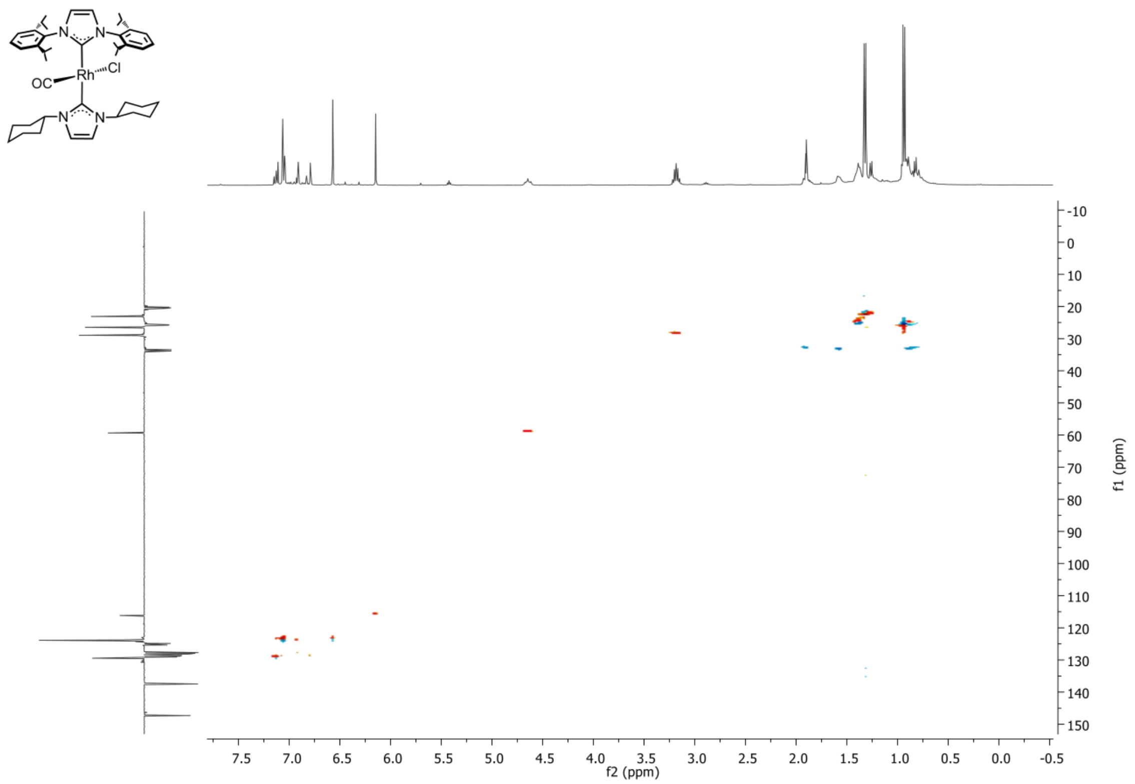

Figure S25. ^{1}H-^{13}C HSQC NMR spectrum of **4b** in toluene-d_8 at 243 K.

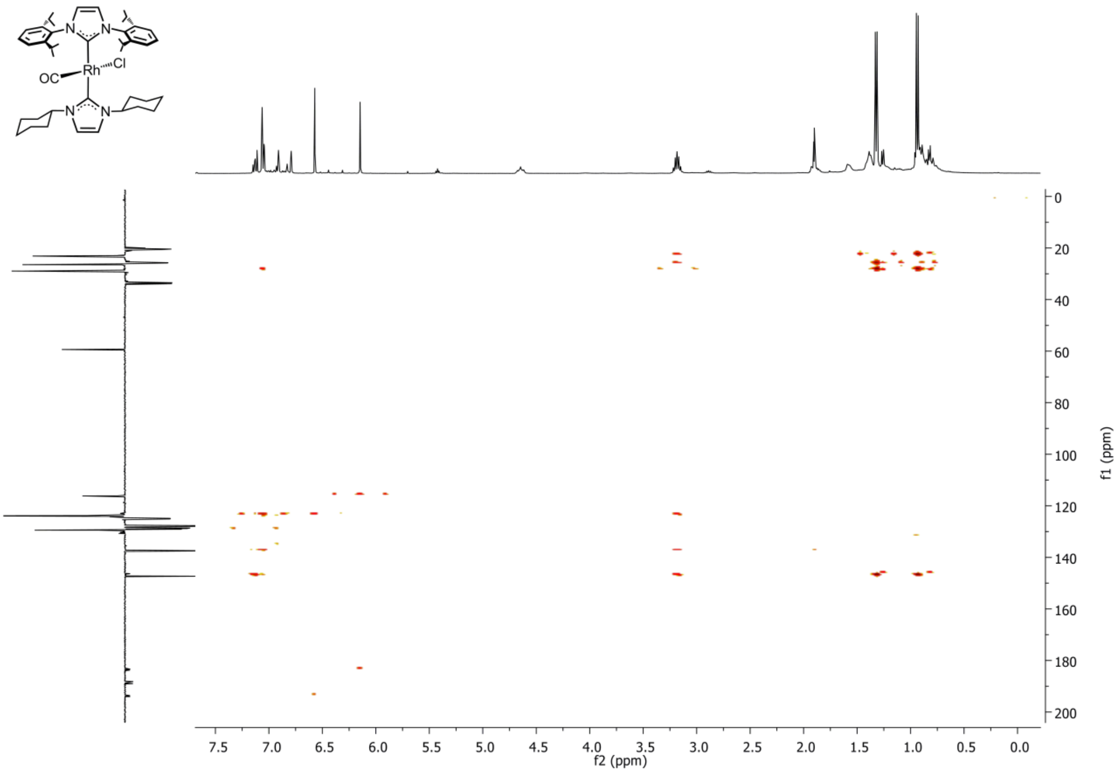

Figure S26. ^{1}H-^{13}C HMBC NMR spectrum of **4b** in toluene-d_8 at 243 K.

RhCl(κ*C*,η^2-BzICoutol)(κ*C*,η^2-ICou^{Bz}) (6)

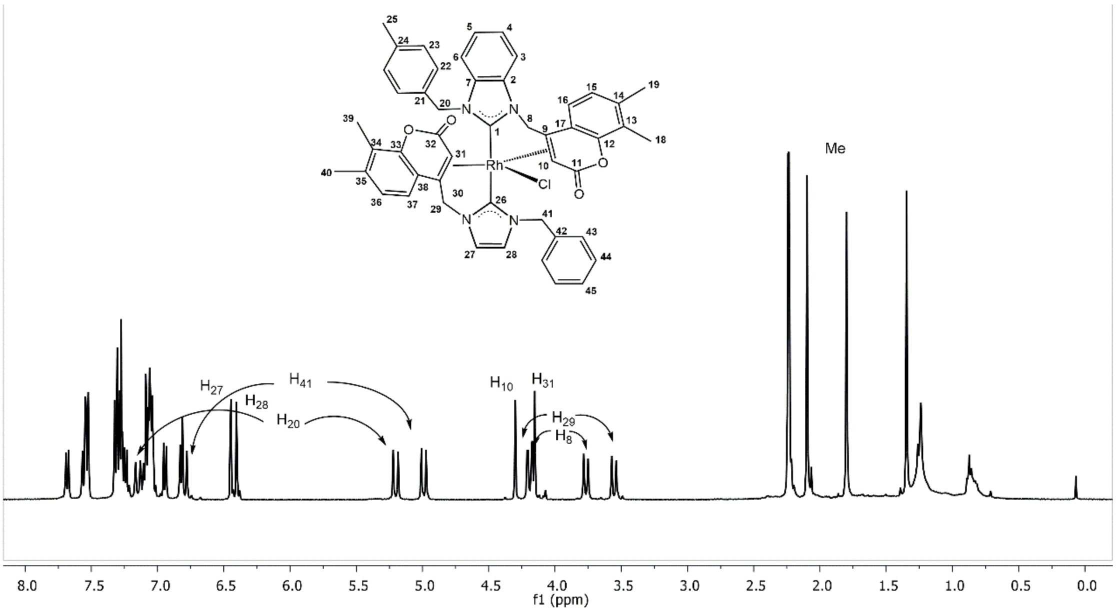

Figure S27. ^{1}H NMR spectrum of **6** in toluene-d_8 at 253 K.

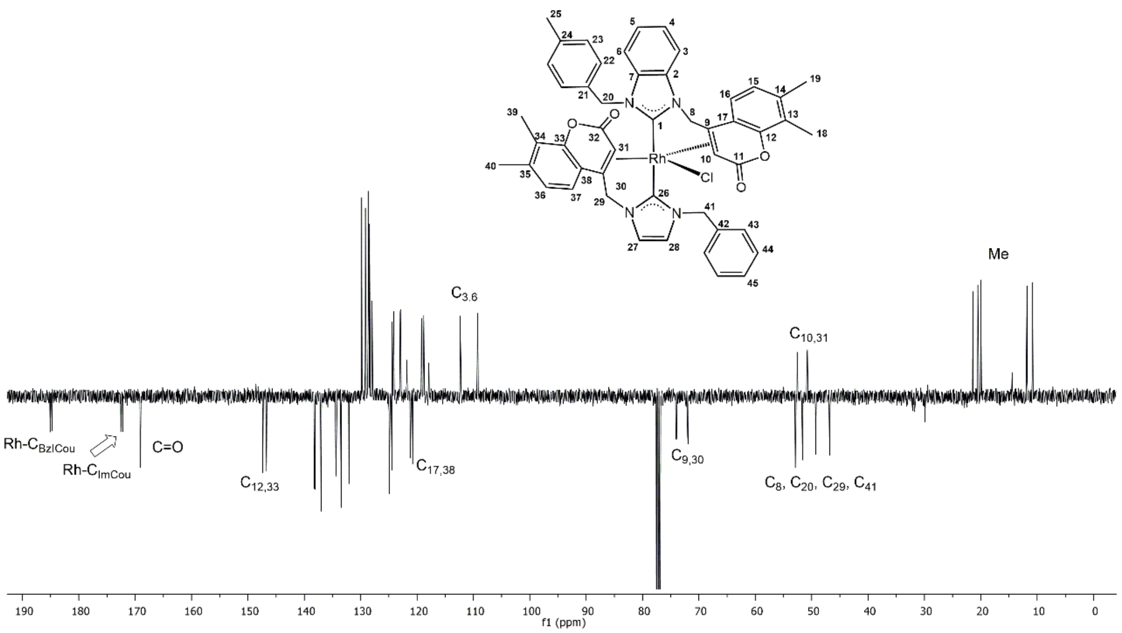

Figure S28. $^{13}C\{^{1}H\}$-APT NMR spectrum of **6** in toluene-d_8 at 253 K.

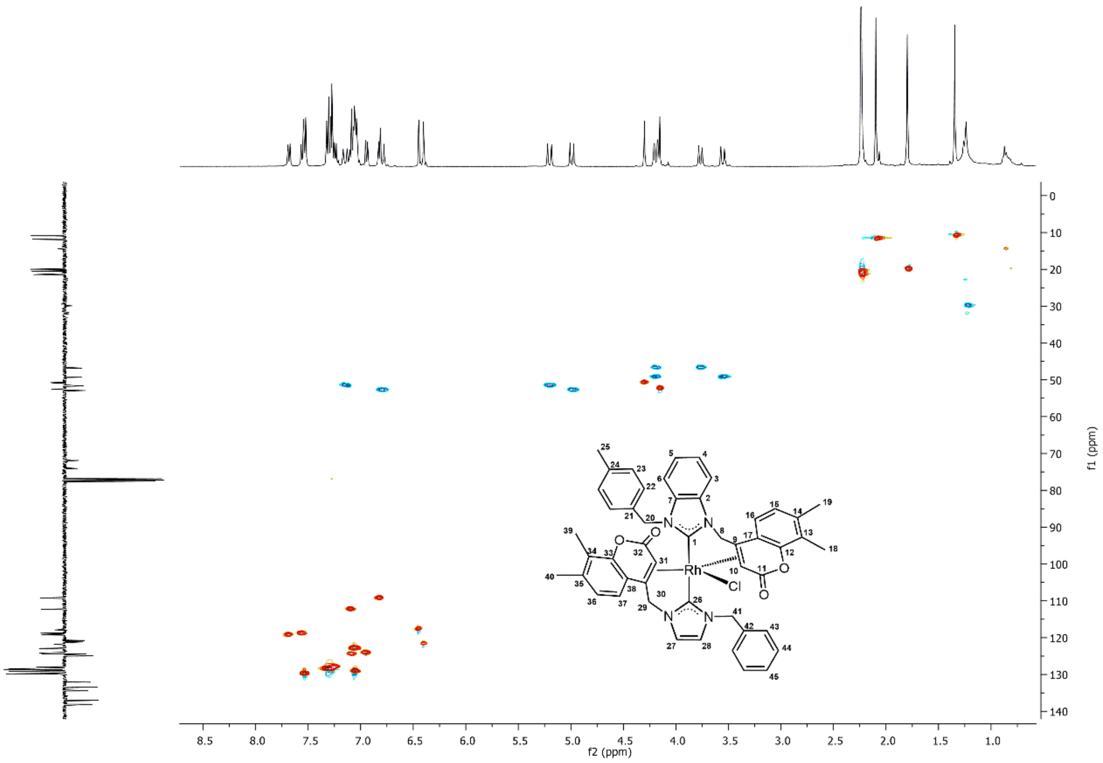

Figure S29. ^{1}H-^{13}C HSQC NMR spectrum of **6** in toluene-d_8 at 253 K.

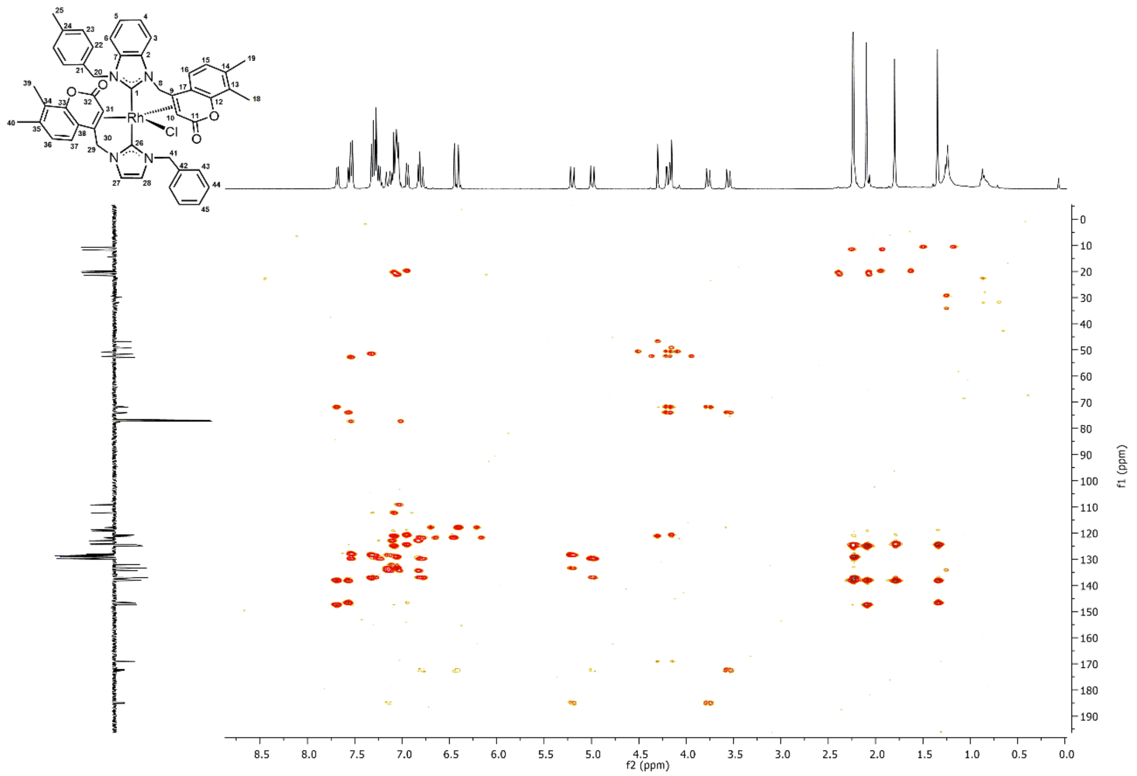

Figure S30. ^{1}H-^{13}C HMBC NMR spectrum of **6** in toluene-d_8 at 253 K.

RhCl(κ*C*,η^{2}-BzICou^{Bu})(IPr) (7)

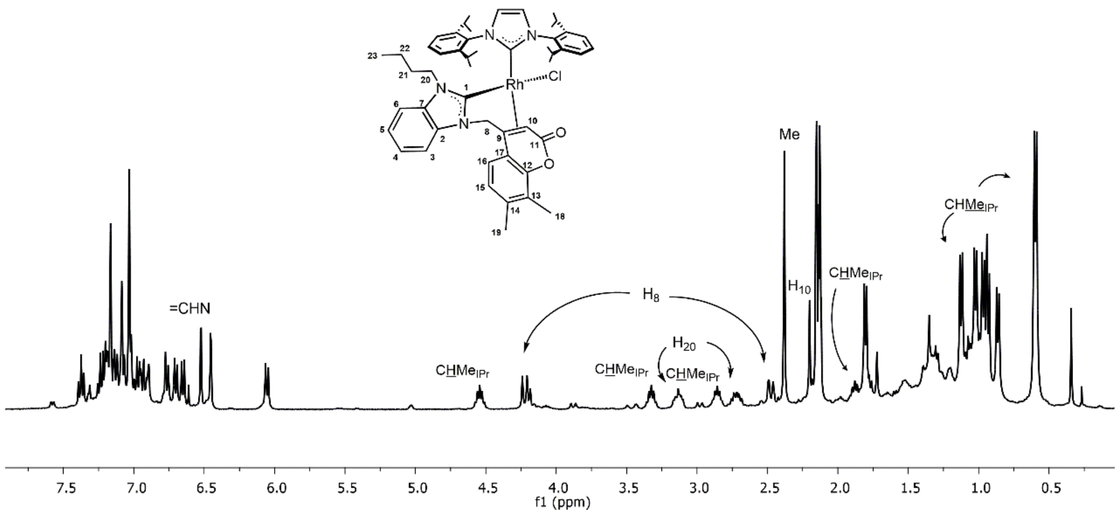

Figure S31. ^{1}H NMR spectrum of **7** in toluene-d_8 at 253 K.

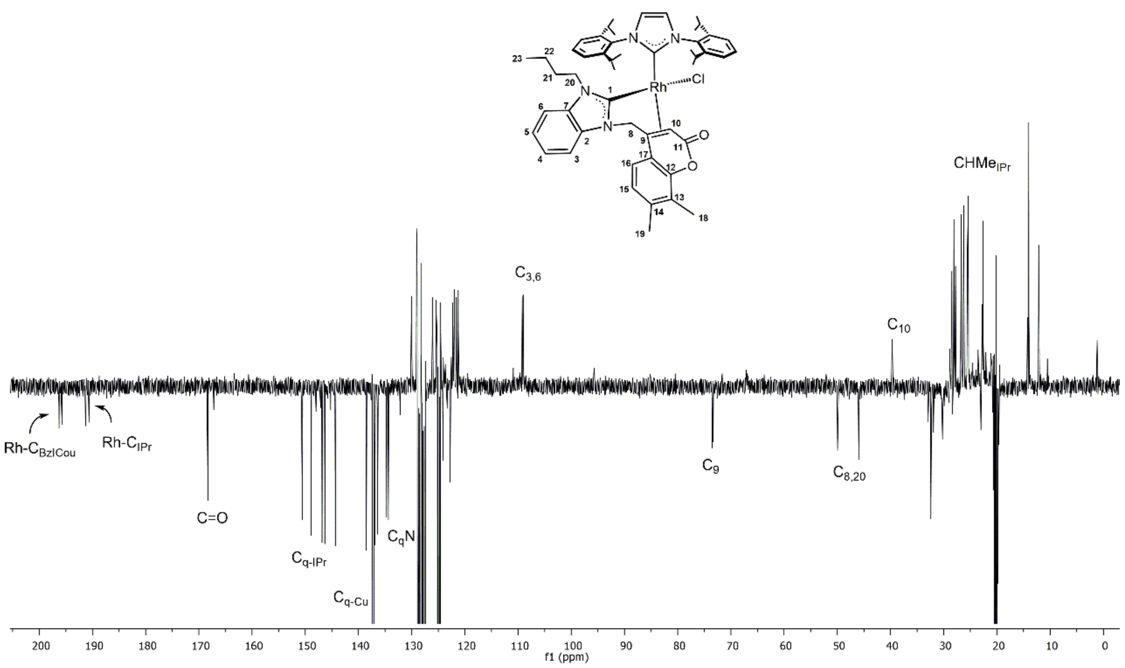

Figure S32. $^{13}C\{^1H\}$-APT NMR spectrum of **7** in toluene-d_8 at 253 K.

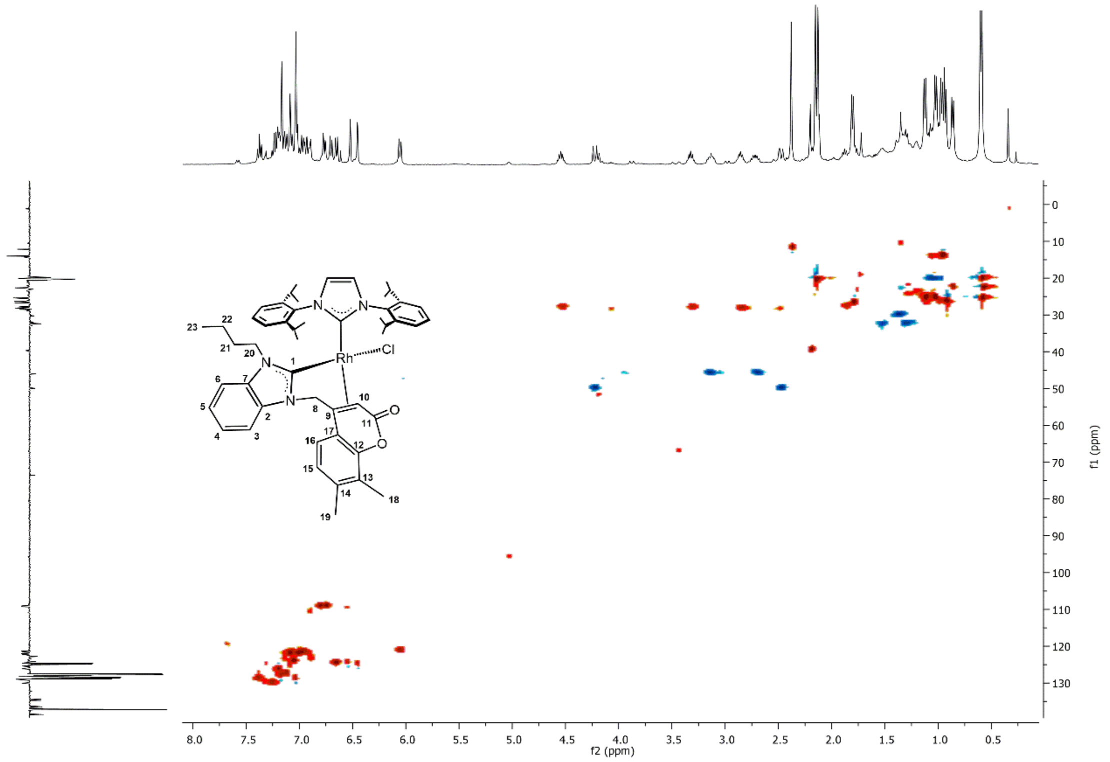

Figure S33. ^1H-^{13}C HSQC NMR spectrum of **7** in toluene-d_8 at 253 K.

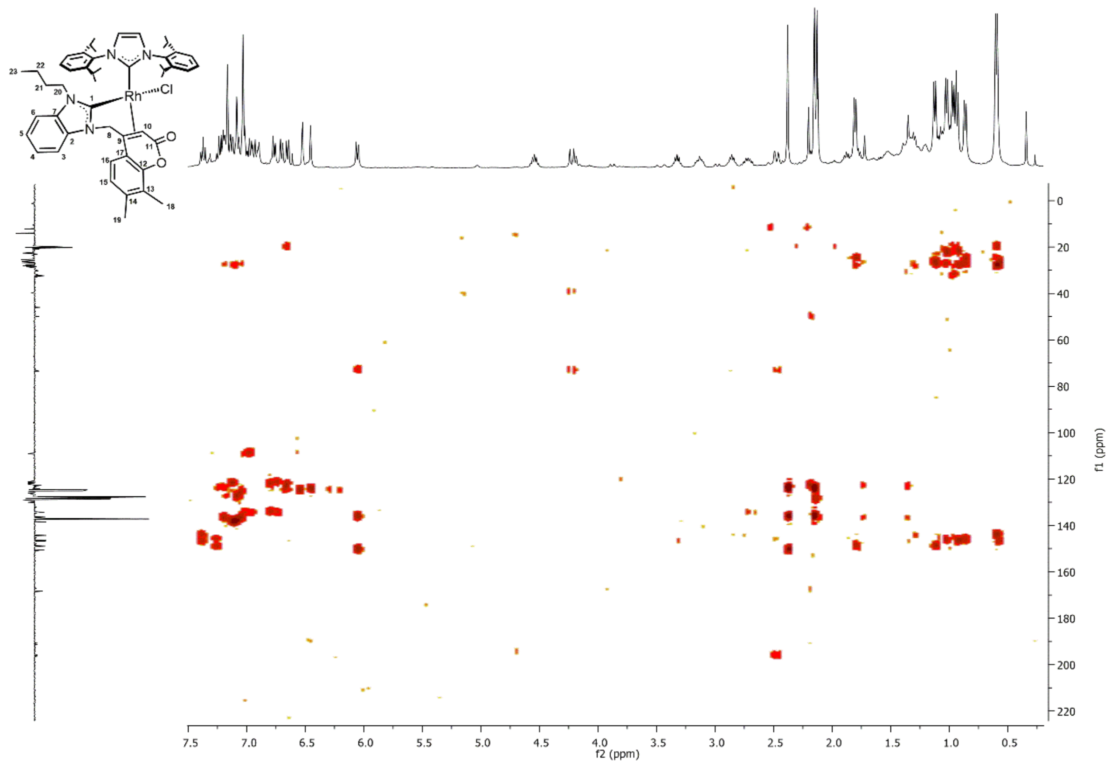

Figure S34. ^{1}H-^{13}C HMBC NMR spectrum of **7** in toluene-d_8 at 253 K.
